# Fabrication of Biodegradable MOF-Based Composites Using Twin-Screw Extrusion for Sustainable Agrochemical Delivery

**DOI:** 10.1021/acsomega.6c03217

**Published:** 2026-06-15

**Authors:** Parimal C. Bhomick, Evdokiya H. Ivanovska, Lila A. M. Mahmoud, Adrian L. Kelly, Valeska P. Ting, Sanjit Nayak

**Affiliations:** † Bristol Composites Institute, School of Civil, Aerospace and Design Engineering, University of Bristol, Bristol BS8 1TR, U.K.; ‡ Research School of Chemistry, 2219 Australian National University, Canberra ACT 2601, Australia; § School of Archaeological and Forensic Sciences, 1905 University of Bradford, Bradford BD7 1DP, U.K.; ∥ School of Chemistry, University of Bristol, Bristol BS8 1TR, U.K.; ⊥ Polymer IRC, School of Engineering, University of Bradford, Bradford BD7 1DP, U.K.; # Bristol Composites Institute, School of Electrical, Electronic and Mechanical Engineering, University of Bristol, Bristol BS8 1TR, U.K.

## Abstract

Twin-screw extrusion can be used for the large-scale preparation of polymer composites as it reduces the use of harsh solvents, provides better dispersibility of the filler material, shortens production times, and improves scalability, compared to the traditional solvent casting method. This study reports the preparation of MOF-based biodegradable polymer hybrid composite membranes using a vertical co-rotating twin-screw extruder for potential application in the delivery of herbicides. Herein, two MOFs, UiO-66 and UiO-66-NH_2_, were synthesized through the solvothermal method and postsynthetically loaded with the three herbicides, 2,4-dichlorophenoxyacetic acid (2,4-D), 2-methyl-4-chlorophenoxyacetic acid (MCPA), and glyphosate. The herbicide-loaded MOFs were incorporated into polycaprolactone (PCL) at 10, 20, and 30 wt % using twin-screw extrusion. The membranes were characterized using Fourier-transform infrared (FTIR) spectroscopy, X-ray diffraction (XRD), scanning electron microscopy (SEM), and thermogravimetric analysis (TGA). Mechanical testing revealed that the incorporation of MOFs enhanced the Young’s modulus of the PCL composite films. The Young’s modulus of the herbicide-loaded MOF-PCL (herbicide@MOF@PCL) composite films increased with increasing percentage of MOFs, as compared to when herbicides were directly loaded into the PCL to prepare the composite films. Herbicide release studies showed that herbicide@MOF@PCL led to sustained release of herbicides. These findings highlight the preparation of MOF-based composite membranes using twin-screw extrusion as an environmentally sustainable and effective method and their potential application in agrochemical delivery.

## Graphical abstract

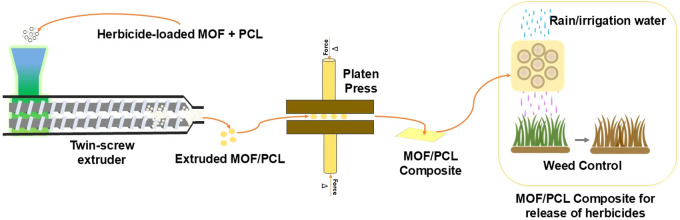

## Introduction

Escalating global food demand has led to the widespread use of agrochemicals, which presents a complex duality. Despite their effectiveness in enhancing agricultural output, the environmental fates of these chemicals and their breakdown products are of serious concern. The accumulation of these substances pollutes critical resources, such as groundwater, and infiltrates the food chain, with direct consequences for ecosystems and human health, including increased risks of cancer and neurological, immunological, and reproductive disorders.^1–5^ Several agrochemicals have been banned by many countries because of their associated toxicity. However, some of them continue to be heavily used as they are essential to maintain crop productivity.^6–8^ Pesticides are mainly delivered by conventional non-targeted methods such as blanket spraying. These methods use a high initial delivery dose; however, it is estimated that sometimes only 0.1% of the applied pesticides reach the targeted site, and the rest ends up in the environment, contributing to pollution and ecotoxicity.^9–13^ It is therefore essential to develop technologies for delivering pesticides and other agrochemicals in a more controlled manner. This challenge is also aligned with a number of the Sustainable Development Goals (SDGs 2: Zero Hunger, SDG 3: Good Health and Well-being, and SDG 6: Clean Water and Sanitation) set by the United Nations.

In recent years, metal–organic frameworks (MOFs) have received considerable attention for various applications, such as gas storage, separation, catalysis, water harvesting, and drug delivery. The use of MOFs as potential vehicles for agrochemical delivery has also been studied recently with promising results, but this area is still in its infancy.^14–16^ Owing to their high surface area, porosity, and tailorable surface properties, MOFs can be engineered to host different kinds of molecules based on their size and site of interaction. Taking advantage of these properties, such as the interaction with guest molecules via adsorption, MOFs have shown excellent ability for the extraction and removal of agrochemicals,^16^ and therefore, can also potentially act as a material for the sustainable delivery of agrochemicals. However, the typically powdered form of many pristine MOFs restricts their practical implementation in agrochemical delivery applications. This limitation can be tackled by developing hybrid MOF-polymer composites, providing an added advantage, such as easy handling, transportation, and targeted delivery.^17^

The preparation of polymer composites often requires the dissolution of the polymer in organic solvents (such as chloroform, 1,4-dioxane, dimethylformamide, methanol, or ethanol), which are not green and not environmentally sustainable, particularly for large-scale applications. Thus, a method that avoids or reduces the use of such solvents is much preferred. The mechanochemical method (where reactions/processes are facilitated by mechanical force) has emerged as an alternative method for carrying out reactions with no/minimal use of solvents.^18,19^ Extrusion is a continuous processing technique in which materials are mixed and forced through narrow openings. Extrusion works on the basic premise of feeding a prepared paste or powder through a hopper and, depending on the geometry of the die, the finished products can be tailored into different shapes, such as hollow tubes, sheets, strips, or cylinders.^19,20^ There are two common types of extrusion, namely, screw and piston types. The former has one (single), two (twin), or more screws that rotate simultaneously and in parallel, thereby enabling continuous processing. Twin-screw extrusion (TSE) has recently gained much attention in the preparation of composites, where materials are transported along a barrel by two co- or counter-rotating screws in an Archimedean manner. Because the screw design includes mixing and kneading parts, the material is subjected to shearing and compression stresses, which provide strong distributive and dispersive mixing by breaking down agglomerates.^19^ Recently, TSE has been successfully used for large-scale mechanochemical synthesis of MOFs and polymer composites because it reduces the use of harsh solvents, provides higher dispersibility of filler materials, and has shorter production times, and high scalability compared to the solvent casting method.^18,19,21–24^ For example, Farha et al. were able to synthesize 100 g of catalytically active UiO-66-NH_2_ at a rate of 1.4 kg/h using a TSE, which showed higher catalytic activity (*t*_1/2_ ≈ 5 min) toward the hydrolysis of nerve agent stimulant dimethyl 4-nitrophenyl phosphate when compared to ball milling or solvothermal synthesis methods (*t*_1/2_ ≈ 6 min). The higher catalytic activity of the TSE route was due to the good dispersibility of the loosely bound particles in water.^18^ Crawford et al. first reported the mass scale synthesis of Al­(fumarate)­(OH) using TSE with a feed rate of 0.6 kg/h, with a yield 8 times higher compared to traditional solvent-based synthesis.^19^ Harding and Reynolds, in 2014, reported the preparation of a polymer/MOF composite to investigate the release of nitric oxide from S-nitrosothiols. They prepared the polyurethane/Basolite-C300 composite using a co-rotating twin-screw extrusion method and found that the MOF was uniformly distributed throughout the polymer.^22^

Herein, we propose a pilot-scale fabrication of an MOF-based hybrid biodegradable polymer composite membrane using the twin-screw extrusion method. The composite membranes were tested for their potential applications in the controlled release of agrochemicals using three herbicides: 2,4-D, MCPA, and glyphosate. These herbicides were selected based on their use as common active ingredients in most household weedkillers and lawn management products, and their wide use for broadleaf weed management.

This study also investigates the effect of MOFs as a filler material on the mechanical properties of the composites. The two selected MOFs (UiO-66 and UiO-66-NH_2_) were thermally and mechanically stable until their decomposition temperature of ∼400 °C, and these MOFs were mixed with a biodegradable polymer (PCL) well below their decomposition temperatures, at 70 °C.^25–27^ In this work, we successfully demonstrated the use of the twin-screw extrusion method for the scale-up fabrication of herbicide-loaded MOF-PCL composite membranes for potential application as vehicles for the controlled release of the herbicides. This fabrication approach prevents the use of harsh and toxic solvents, which are often used for dissolving polymers in the process of manufacturing composites, reduces production times, and enables easier scale-up of composite production.

## Experimental Section

### Chemical and Reagents

Zirconium­(IV) tetrachloride (98%, anhydrous), terephthalic acid (98%), and 2-aminoterephthalic acid (99%) were purchased from Merck. Poly­(caprolactone) diol (average M.W. 2000) was purchased from Fisher Scientific Chemicals. 2,4-D, MCPA, and glyphosate were purchased from Acros Organics. All reagents and chemicals were of analytical grade and were used without further purification. All solvents were obtained from Fisher Scientific.

### Synthetic Procedures

In this study, two zirconium-based MOFs (UiO-66 and UiO-66-NH_2_) were selected for their superior mechanical, thermal, and chemical stability as well as their environmentally benign nature. These MOFs were used to load the herbicides via the postsynthetic loading (PSL) method. In a typical PSL, each synthesized MOF was first activated with ethanol and dried at 110 °C under vacuum for 24 h prior to loading the herbicides. 2,4-D, MCPA, and glyphosate were loaded into these activated MOFs by immersing them in the respective solutions for 48 h, followed by their incorporation into biodegradable polycaprolactone membranes. These membranes were further studied for the release of the pesticide for 72 hours in an aqueous medium.

### Preparation of UiO-66

UiO-66 was prepared using the method described in our previous work with slight modifications.^28^ To a 40 mL Teflon-lined glass vial, 0.63 g (2.703 mmol) of zirconium tetrachloride (ZrCl_4_) and 0.898 g (5.408 mmol) of benzene dicarboxylic acid (BDCA) were added, followed by the addition of 15 mL of dimethylformamide (DMF) and 0.5 mL of conc. HCl. The vials were tightly closed using the Teflon-lined cap, and the mixture was sonicated for 20 min at 25 °C and placed into a programmable oven at 120 °C for 24 h with a heating ramp of 10 °C per minute from room temperature (20 °C), followed by cooling to 25 °C at a rate of 2 °C per minute. The white cloudy suspension was then centrifuged (4500 rpm) for 10 min to obtain a white precipitate. The white solid was washed and left in fresh DMF for 24 h, which was repeated three times, to remove any unreacted species, and then soaked in ethanol (30 mL × 3 times for 30 min each) before filtering and drying in a vacuum oven for 24 h at 70 °C.

### Preparation of UiO-66-NH_2_

UiO-66-NH_2_ was prepared using the method described in our previous work with slight modifications.^28^ ZrCl_4_ (0.63 g, 2.703 mmol) was added to 5 mL of DMF and 0.5 mL of HCl in a 40 mL Teflon-lined glass vial, and the mixture was sonicated for 20 min at 25 °C. To the resulting mixture, 0.979 g (5.408 mmol) of 2-aminobenzenedicarboxylic acid and 10 mL of DMF were added and sonicated for another 20 min at 25 °C. The vial was tightly closed using a Teflon-lined cap and placed in a programmable oven at 120 °C for 24 h at a heating rate of 10 °C per minute and a cooling rate of 2 °C per minute until the oven temperature dropped to 25 °C. The resultant pale-yellow cloudy suspension was centrifuged for 10 min at 4500 rpm. The resulting pale-yellow solid was washed thoroughly with fresh DMF (three times, 24 h each time) to remove any unreacted species and then soaked in ethanol (30 mL × 3 times for 30 min each) before filtering and drying in a vacuum oven for 24 h at 70 °C.

### Loading of Herbicides

In two 100 mL volumetric flasks, stock solutions were prepared by separately dissolving 1.33 g (6.017 mmol) of 2,4-D, 1.20 g (5.981 mmol) of MCPA, and 1.00 g of glyphosate (5.914 mmol) in 100 mL of ethanol. For loading the herbicides, 10 mL of the herbicide solution was transferred to a 30 mL glass vial, and 0.05 g of pristine MOF was added. The mixtures were stirred at 600 rpm for 48 h at room temperature. The herbicide-loaded MOF was recovered by centrifugation (10 min at 4500 rpm) and washed with ethanol (3 × 10 mL). The resulting two herbicide-loaded MOFs (herbicides@MOF) were dried in a vacuum oven for 24 h at 80 °C. The loading capacity of the MOFs was calculated using the method described in section S6 of Supporting Information.

### Preparation of the Herbicide-Loaded MOF-PCL Composite

The herbicide@MOF polymer composite was fabricated by current industrial extrusion processing methods. MOFs were incorporated into PCL using a co-rotating twin-screw extruder. A Pharma11 HME co-rotating twin-screw extruder (Thermo Fisher Scientific) was used with the die section removed to allow for the direct collection of granulated material exiting the barrel. The extruder screw speed was maintained at 50 rpm, and the barrel temperature was adjusted to 70 °C using the control panel on the instrument. In a typical extrusion procedure, prior to compounding, the loaded MOFs were dried in a vacuum oven at 80 °C, as described in the previous section, and the PCL pellets were dried separately in an oven at 40 °C. PCL was mixed with different percentages of herbicide@MOF (10, 20, and 30%) by gently grinding the materials using a mortar and pestle. The mixture was then manually fed into the extruder at 70 °C. The extruded pellets (see Figure S1 in the Supporting Information) were collected directly without a die and conditioned for an hour at room temperature. The pellets were then made into a thin film (see Figure S1) using an automatic hydraulic press (Fontjine platen press) by pressing the pellets between two brass plates and heating them to 70 °C at a pressure of 6 kN for 4 min, followed by subsequent pressures of 30, 60, 100, and 150 kN, each for 45 s. The temperature was set to 25 °C by using a cooling unit (RA50-Trop M305, Frigosystem) before removing the composite films.

### Release Studies

To test for herbicide release profiles, 5 mg of herbicide@MOF and PCL composites (cut into 2 × 2 cm pieces) were submerged in 30 mL of distilled water for up to 72 h at room temperature. The filtered aliquots were analyzed using a UV–visible spectrophotometer to quantify the concentration of herbicides released from the MOFs and their PCL composites.

### Material Characterization

Fourier-transformed infrared (FTIR) spectra of the samples were recorded over the range of 650–4000 cm^–1^ using a PerkinElmer Spectrum 100 FTIR spectrometer fitted with a PerkinElmer Universal ATR sampling device. Thermogravimetric analyses (TGA) were carried out using a Q5000IR thermogravimetric analyzer (TA Instruments). Samples (ca. 5 mg) were heated in a platinum pan from 30 to 800 °C at 5 °C min^–1^ under a nitrogen purge gas flow of 25 mL min^–1^. The data were processed and analyzed using the TA Instruments Universal Analysis 2000 software. SEM images and energy dispersive X-ray (EDX) elemental analysis data were collected using an FEI Quanta 400 E-SEM instrument fitted with an Oxford Xplore30 EDS system. Powder X-ray diffraction (PXRD) data were collected at ambient temperature using a Bruker D8 diffractometer with Cu Kα1,2-radiation (λ = 0.154018 nm, 1600 W) source. Mechanical testing was performed using a Universal testing machine (INSTRON 5568 uniaxial test machine). The tensile specimen was ISO 37-2, which had a neck thickness of 5 mm, a parallel section length of 25 mm, and a total length of 48 mm. The specimens were stamped using a sample cutter, and a grip separation of 100 mm/min was applied during the test, from the initial gauge length of 25 mm until the sample broke. Five specimens were tested for each measurement, and the average values, along with the corresponding mean values of the standard errors, were calculated.

## Results and Discussion

### Characterization of MOFs

All pristine and herbicide-loaded MOFs and their hybrid composite membranes were characterized using FTIR spectroscopy, TGA, PXRD, SEM, and elemental mapping. The powder X-ray diffraction (PXRD) patterns of the pristine MOFs, herbicide@MOF, and their polymer composites are shown in Figure 1. The diffraction patterns for both UiO-66 and UiO-66-NH_2_ match the patterns reported in literature,^29^ which indicates the successful synthesis of the MOFs. Upon loading the herbicides (2,4-D, MCPA, and glyphosate), there was no significant change in the two sharp peaks at 2θ ∼ 7.3 and 8.4° for the two MOFs, indicating that the 2,4-D, MCPA, and glyphosate-loaded MOFs were stable and the crystallinity of the MOFs was retained after loading (Figure S2). However, slight shifts of around 0.1–0.2% for 2θ 7.3° and ∼8.4° for herbicide-loaded MOFs could be observed. These arise from minor lattice strain or contraction in the UiO-66 frameworks induced by herbicide–MOF interactions, such as the coordination to Zr sites or H-bonding to linkers. For the PCL composites, these characteristic peaks (at 2θ ∼7.3 and 8.4°) are observed, indicating the presence of crystalline MOFs inside the PCL matrix. For the PCL, the three well-defined peaks at 2θ = 21.2° [(100) plane], 22.0° [(111) plane], and 23.5° [(200) plane] (see Figure S2) are in agreement with the literature, indicating an orthorhombic crystal lattice of PCL.^30^

**Figure 1:**
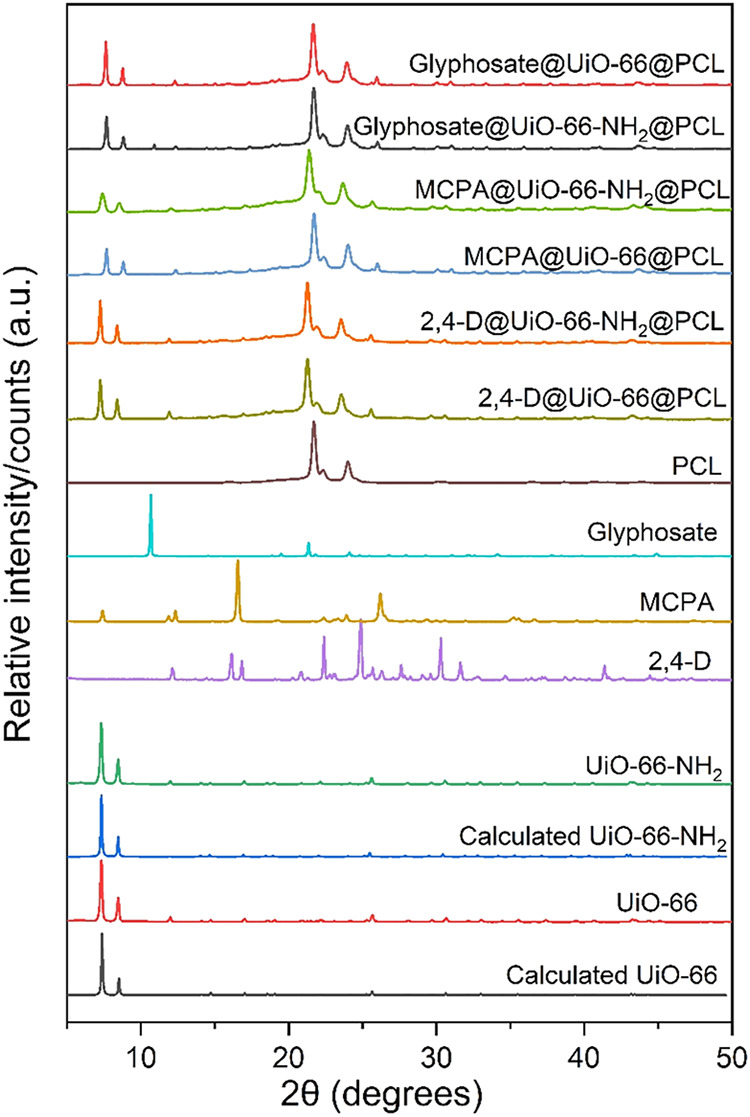
PXRD patterns of UiO-66, UiO-66-NH_2_, and their PCL composite loaded with 2,4-D, MCPA, and glyphosate.

FTIR studies were performed to understand the surface functionality of the pristine MOFs and their polymer composites, and the relevant spectra are shown in Figure 2. The FTIR spectra of pristine UiO-66 and UiO-66-NH_2_ are in good agreement with those reported in the literature.^31,32^ The bands at 1655 and 1580 cm^–1^ for the synthesized UiO-66 correspond to the asymmetric O–C=O, and the band centered around 1381 cm^–1^ appears due to the symmetric stretching of the C–O bond arising from the carboxylate group of the organic linker. The characteristic bands at 740 and 654 cm^–1^ indicate the bending of the O–H group from the BDC linker and the stretching of the Zr–O bond from the metal ion cluster. For UiO-66-NH_2_, the band at 1570 cm^–1^ corresponds to the bending vibration of N–H, and the band at 1434 cm^–1^ appears from the C=O stretching of the carboxylate group coordinated to Zr (IV). The bands at 1380 and 1257 cm^–1^ correspond to the C–N bond stretching vibrations of NH_2_ in the organic linker. The FTIR spectra also provide information on the loading of 2,4-D, MCPA, and glyphosate (Figure S3) into the MOFs. Bands centered at around 1476, 1228, and 1047 cm^–1^ for pure 2,4-D can also be observed in 2,4-D-loaded UiO-66 and UiO-66-NH_2_. Similarly, for MCPA-loaded UiO-66 and UiO-66-NH_2_ and their PCL composites, the appearance of characteristic bands at around 1490, 1296, and 1135 cm^–1^ for pure MCPA was identified in the MCPA-loaded MOFs. In the case of glyphosate, bands centered around 1334 and 1070 cm^–1^ represent characteristic peaks for glyphosate, originating from the stretching vibrations of carboxylate and the symmetric stretching of P–O bonds of the phosphonate group, respectively.^33^ It was also observed that upon loading the glyphosate onto the MOFs, the glyphosate peak at 1334 cm^–1^ was partly masked by the peak around 1375–1380 cm^–1^, which originated from the MOF, and slightly shifted to a higher wavenumber of 1390 cm^–1^. This slight shift toward a higher wavenumber and increase in intensity could be due to the supramolecular interaction between the MOF and the functional groups of glyphosate.^34,35^ These changes in the FTIR spectra indicate that the three herbicides were successfully loaded into UiO-66 and UiO-66-NH_2_. FTIR studies of the PCL composites also show characteristic bands at 2942 cm^–1^ for the C–C bond stretching and a sharp peak at 1720 cm^–1^ resulting from the C=O stretching of polycaprolactone. It can also be noted that the spectra of herbicide@MOF@PCL composites are highly similar. This is due to the high amount of PCL (90% of PCL) in the composites as compared to the MOFs. Thermogravimetric analyses (TGA) of all samples were performed (Figure 3). All MOFs show three weight loss steps. The first weight loss at around 150 °C (Figure S4) can be attributed to the loss of water from the pores of the synthesized materials. The second decomposition starting from 150 to 300 °C is due to the decomposition of the organic linker and slight loss of water due to the dehydroxylation of Zr-oxo clusters.^36,37^ Similar TG patterns have been reported in previous work on UiO-66 using TG-MS by Athar et al. on UiO-66 samples.^38^ The 2,4-D, MCPA, and glyphosate-loaded MOFs exhibit another step of weight loss between 175 and 380 °C, which is attributed to the decomposition of the herbicides.^39,40^

**Figure 2:**
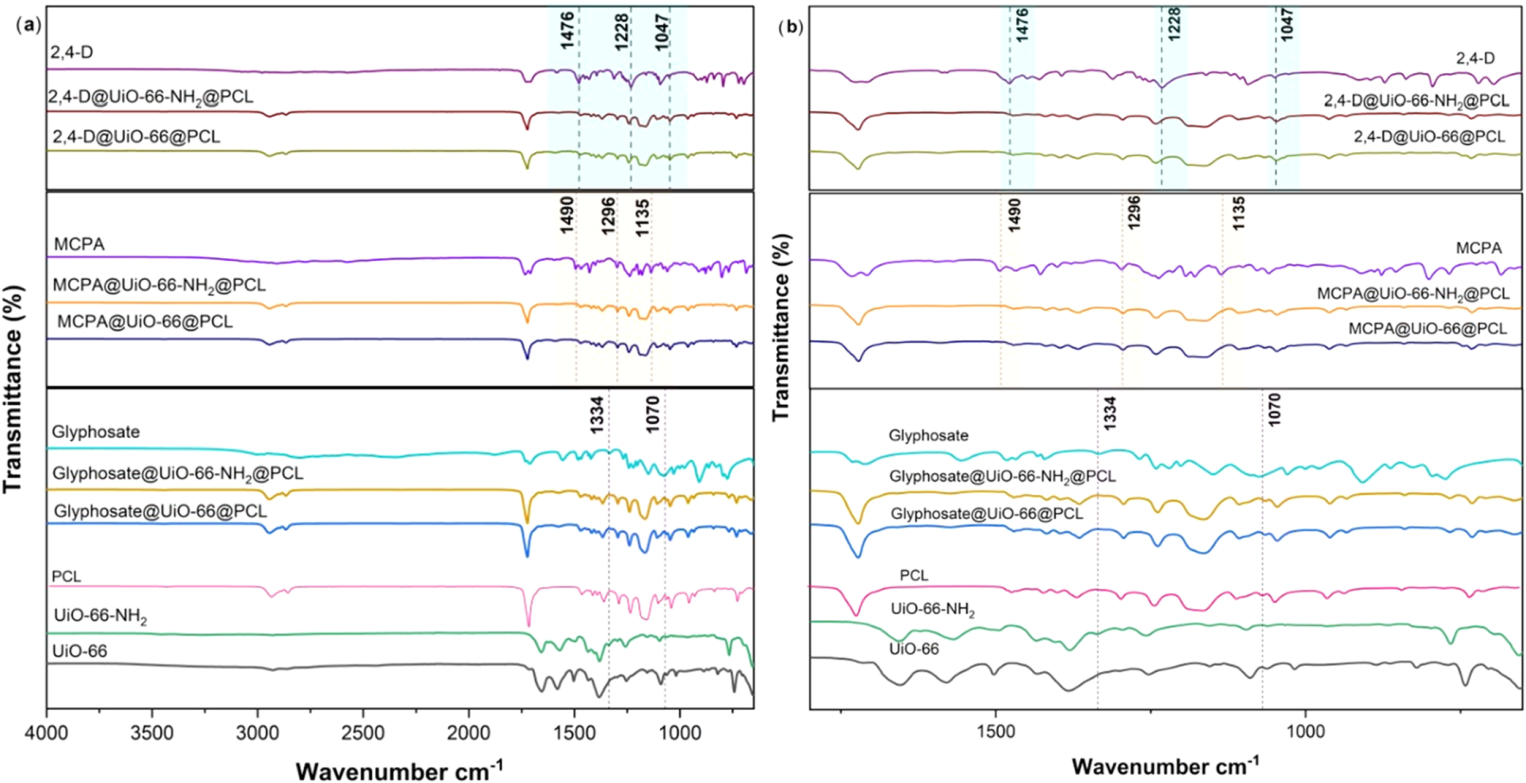
(a) FTIR spectra of herbicide-loaded UiO-66 and UiO-66-NH_2_ PCL composites, along with FTIR spectra of pristine UiO-66, UiO-66-NH_2_, and herbicides, (b) Inset of (a) from 1500 to 650 cm^–1^.

**Figure 3:**
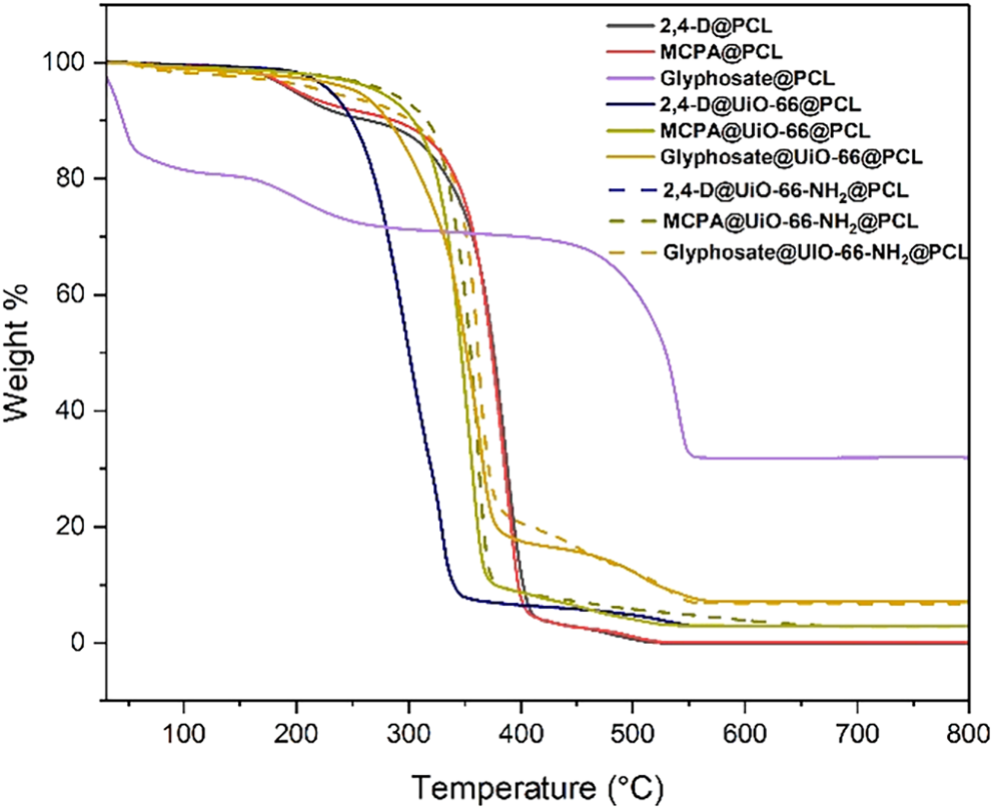
TGA thermograms of herbicide@PCL, herbicide@UiO-66@PCL, and herbicide@UiO-66-NH_2_@PCL composites.

The final weight loss occurs due to the total degradation of the MOF and loaded herbicides, which occurs between 400 and 600 °C. For PCL loaded directly with the herbicides, the degradation patterns of 2,4-D@PCL and MCPA@PCL were in perfect agreement. However, for glyphosate@PCL, there is an initial weight loss at around 100 °C, which could be due to the presence of impurities (residual water from crystallization, phosphoric acid, formic acid, and n-nitrosoglyphosate).^41,42^ There is a second decomposition at around 190–220 °C, which is attributed to the mass loss of impurities in the glyphosate sample, as also observed by Chen et al., who reported a similar degradation pattern for glyphosate in earlier studies.^40^ The third decomposition starting at around 350 °C for glyphosate@PCL can be attributed to the degradation of glyphosate metabolites.^40^ For the herbicide@MOF@PCL composites (Figure 3b,d), TGA showed a significant loss in weight percentage starting at around 230 °C because of the thermal degradation of PCL and a second slight weight loss at around 490 °C, which is due to the thermal degradation of the herbicides and thermal degradation of MOFs, including organic ligands (terephthalic acid or 2-aminoterephthalic acid) and zirconium clusters in the polymer matrix.^43^ It can also be seen that the thermal stability of 2,4-D@MOF@PCL and MCPA@MOF@PCL composites dropped slightly with the addition of MOFs, which could possibly be due to the catalytic depolymerization by the unsaturated metal sites of the MOF. Thus, thermal analysis of the materials revealed multistep thermal degradation for MOFs and their herbicide-loaded PCL composites, with additional decomposition stages due to herbicide and PCL interactions, and a slight reduction in composite stability attributed to the catalytic effects of MOF metal sites.

The surface morphologies of the pristine MOFs and their composites were analyzed by scanning electron microscopy, and the results are shown in Figure 4. The morphology of the pristine MOFs is homogeneous and without impurities that sometimes appear from the extruder or from the brass plates used during hot pressing. The results are also in good agreement with the PXRD data, which indicates a single phase of the materials. SEM imaging of the PCL composites showed well-dispersed herbicide@MOF in the matrix of the PCL polymer (see Section 5, Figure S5 for elemental mapping images in Supporting Information). However, while examining the direct loading of herbicides into the PCL matrix, it was found that the herbicides were not uniformly distributed throughout the polymer matrix (Figure S5).

**Figure 4:**
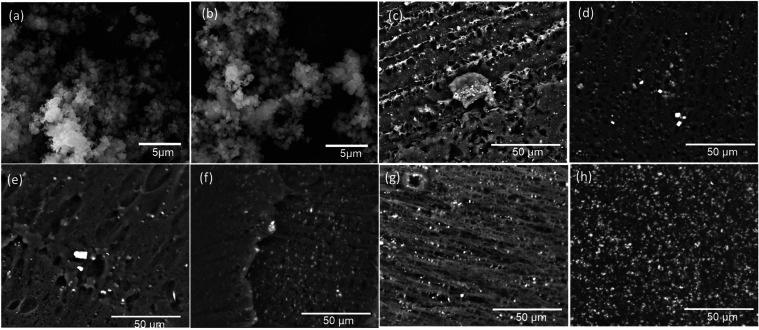
SEM images of (a) UiO-66, (b) UiO-66-NH_2_, (c) 2,4-D@UiO-66@PCL, (d) 2,4-D@UiO-66-NH_2_@PCL, (e) MCPA@UiO-66@PCL, (f) MCPA @UiO-66-NH_2_@ PCL, (g) glyphosate@UiO-66@PCL, and (h) glyphosate@UiO-66-NH_2_@ PCL.

### Mechanical Properties

For practical applications in agrochemical delivery, the mechanical properties of the composites play important roles. PCL composite films loaded with different herbicide@MOFs (10, 20, and 30%) were tested for their mechanical properties. The tensile stress and Young’s modulus of the PCL@MOF composites are shown in Figure 5.

**Figure 5:**
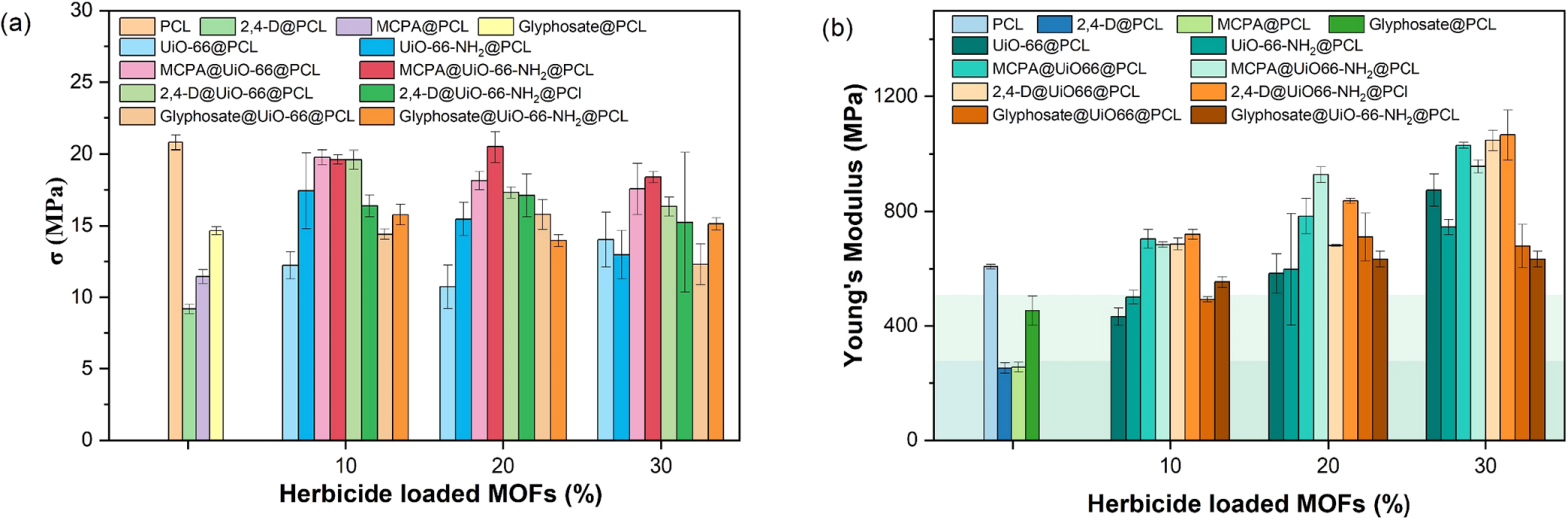
Mechanical properties of PCL@MOF composites: (a) tensile stress, (b) Young’s modulus of pristine PCL, MOF@PCL, herbicides@PCL, and MOF@PCL composites at different loading percentages.

The tensile stress and Young’s modulus for PCL at zero slope were found to be 20.78 ± 0.51 MPa and 606.89 ± 8.22 MPa, respectively. The addition of herbicides directly into the PCL decreased both the tensile stress and Young’s modulus. Additionally, it was also observed that the herbicides@PCL composite became sticky during the extrusion process and quickly became very brittle (within a week) once transformed to a sheet (Figure S1). This could be caused by the moderately acidic characteristics (p*K*_a_ of ∼2 to 3) of all three herbicides. A plausible mechanism is that upon deprotonation, the herbicides attack the carbonyl carbon of PCL’s ester linkage, initiating chain scission and leading to the embrittlement of the PCL composites. Anaya-Mancipe et al. also reported similar findings on the hydrolytic degradation of PCL in an acidic environment.^44^ Mechanical testing was carried out at 10, 20, and 30% loadings of the herbicides@MOF in the PCL matrix, produced via twin-screw extrusion (10 g scale). It was observed that the tensile stress of all the composite films decreased upon the formation of the composite compared to the pure PCL film. However, the tensile strength of all the composites in this work is higher than that of the tensile strength of UiO-66 (0.40 MPa) and UiO-66-NH_2_ (4.18 MPa), as observed in an earlier study by Dhainaut et al.^45^ On the other hand, the Young’s modulus of the PCL@MOF composites increased with increasing loading percentage of MOFs. The increase in Young’s modulus upon the addition of MOFs to PCL is possibly due to the coordination of the ester group of PCL with the Zr-metal node of UiOs.^46^ This is also due to the high density of covalent bonds per sectional area and the hydrogen bonding between MOFs and PCL, which stiffen the composite films. Thus, with an increase in the percentage of MOFs, Young’s modulus continues to increase.

### Herbicide Loading and Release

The loading capacities of the three herbicides were estimated by suspending the loaded MOFs in distilled water and sonicating for 60 min. The loading capacities were found to be 21.06, 24.41, and 17.33% for 2,4-D@UiO-66, MCPA@UiO-66, and glyphosate@ UiO-66, respectively. The loading capacities were found to be 23.91, 27.08, and 19.75% for 2,4-D@UiO-66-NH_2_, MCPA@UiO-66-NH_2_, and glyphosate@UiO-66-NH_2_, respectively. To test the herbicide release profiles, each herbicide@PCL sample and its corresponding herbicide@MOF@PCL composite (2 cm × 2 cm) was submerged in 30 mL of distilled water for up to 72 h at room temperature. The filtered aliquots were analyzed using a UV–visible spectrophotometer to quantify the concentration of herbicides released from the composite membrane. When herbicides were directly incorporated into PCL to form herbicide@PCL, the amounts of 2,4-D, MCPA, and glyphosate released daily were rapid (see Figure S7). This could be attributed to the weak cross-linking of the herbicides to the PCL matrix and composite embrittlement, as discussed earlier. PCL composites loaded with different percentages of herbicide@MOF (10, 20, and 30%) were also investigated in their release studies. The results are shown in Figure 6.

**Figure 6:**
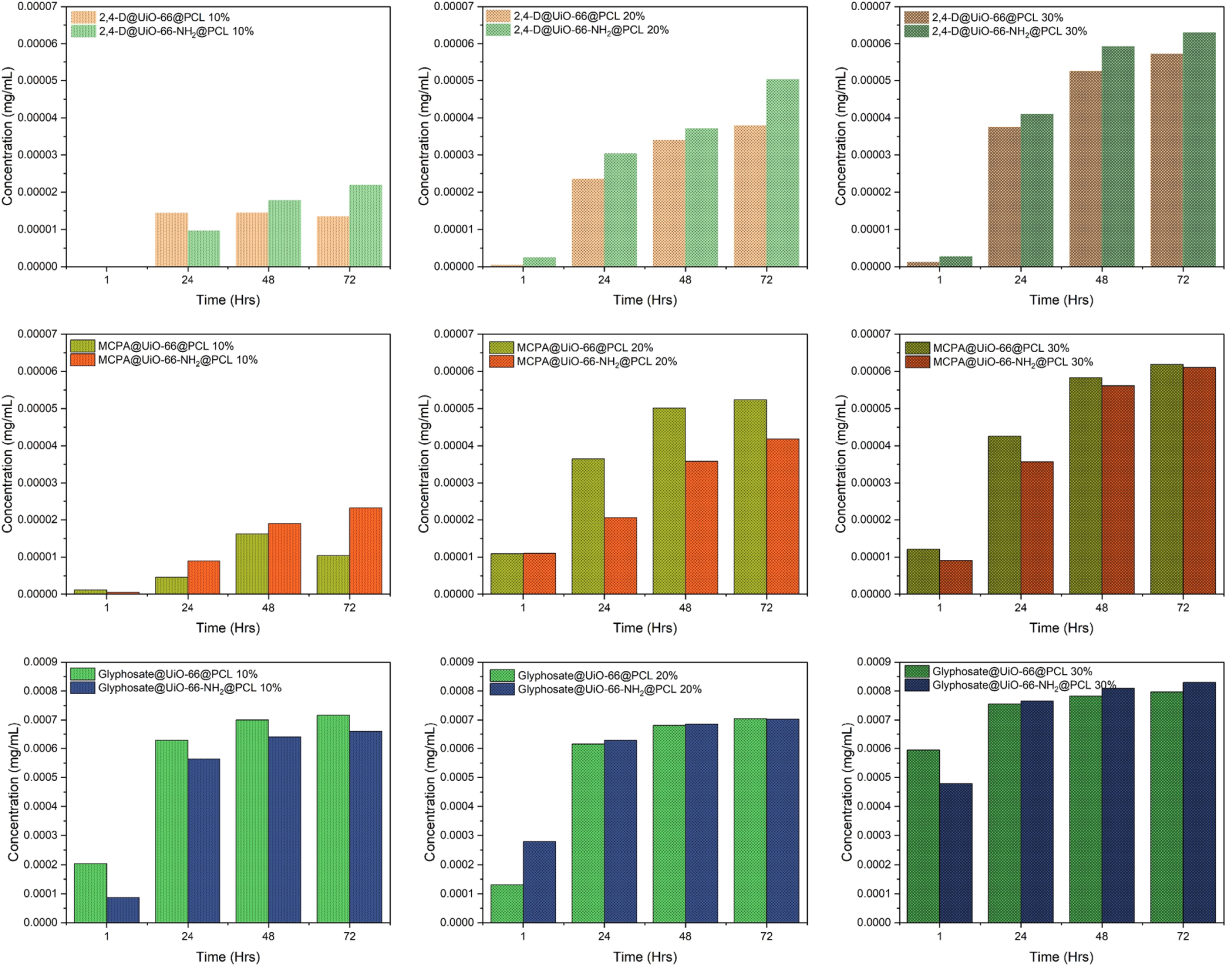
Release studies of herbicides from MOF@PCL composites (release study was performed with a 2 × 2 cm piece of herbicide@PCL composite submerged in 30 mL of water in a closed tube).

The incorporation of the herbicide@MOF within the PCL matrix leads to a sustained release of the herbicides. The release of 2,4-D from 2,4-D@UiO-66@PCL and 2,4-D@UiO-66-NH_2_@PCL was found to increase over time, with 2,4-D@UiO-66-NH_2_@PCL releasing slightly higher than UiO-66. This may be because amino-functionalized UiO-66 forms hydrogen bonds with the −Cl or −COOH groups of the 2,4-D molecules.^31^ For MCPA from MCPA@UiO-66@PCL and MCPA@UiO-66-NH_2_@PCL, with increasing percentages of MOFs, there was an increase in the release of MCPA, but no trend among the two MOFs was found for 2,4-D. Nevertheless, the herbicide release for 2,4-D@MOF@PCL and MCPA@MOF@PCL increases as the percentage of loading increases. This was expected because the amount of herbcide@MOF in the PCL matrix increased with increased MOF loading. Even for glyphosate@MOF@PCL, the release of glyphosate followed the trend of 2,4-D and showed a slight increase in release, with increasing loading percentages of MOFs, with glyphosate@UiO-66-NH_2_@PCL releasing slightly higher amounts of glyphosate at 20 and 30%, respectively. Overall, this suggests that twin-screw extrusion can be potentially used as a tool to scale-up MOF-based composites for the controlled and efficient release of herbicides without the use of harsh solvents, as used in the solvent casting method.

## Conclusions

In conclusion, this study successfully demonstrated a solvent-free route for the preparation of MOF-based PCL composites for the controlled release of herbicides using the twin-screw extrusion method. The process was successfully scaled up to a 10 g scale using a twin-screw extruder, indicating the potential for large-scale production for practical applications. The findings reveal that incorporating herbicides into the MOF framework prior to embedding them into the PCL matrix significantly enhances the herbicide release efficiency and mitigates composite degradation. Direct herbicide loading into the PCL matrix, in contrast, resulted in embrittlement and rapid material degradation within a few days. TGA, XRD, SEM, mechanical testing, and cross-linking studies further validated the stability and performance of the herbicides@MOF@PCL system. The findings also revealed that UiO-66-NH_2_ is a more efficient MOF than UiO-66 because of its ability to sustainably release herbicides. These promising results underscore the potential of UiO-66-based (especially UiO-66-NH_2_) PCL composites prepared using twin-screw extrusion methods for broader applications, including the development of other MOF-based composite systems for various agrochemical delivery purposes.

## Supplementary Material



## Notes

Additional camera images, PXRD plots, IR spectra, TGA plots, SEM images, elemental mapping, mechanical property data, and release study plot (PDF)

## Abbreviations

2,4-D: 2,4-dichlorophenoxyacetic acid
IR: infrared
MCPA: 2-methyl-4-chlorophenoxyacetic acid
MOF: metal–organic framework
PCL: polycaprolactone
PXRD: powder X-ray diffraction
SEM: scanning electron microscope
TGA: thermogravimetric analysis
