# Supplementary material for: Fabrication of Biodegradable MOF-Based Composites Using Twin-Screw Extrusion for Sustainable Agrochemical Delivery
Source: ACS Omega. 2026 Jun 15;11(25):37927–35. doi: 10.1021/acsomega.6c03217 (PMC13325368; doi:10.1021/acsomega.6c03217)
Supplement: Supplementary file 1 [file ao6c03217_si_001.pdf]

Electronic Supplementary Information

Fabrication of Biodegradable MOF-based Composites using Twin Screw Extrusion for Sustainable Agrochemical Delivery

Parimal C. Bhomick[1,2,†], Evdokiya H. Ivanovska[3,†], Lila A. M. Mahmoud[4], Adrian L. Kelly[5], Valeska P. Ting[,2,6], and Sanjit Nayak*[,1]*

[1]Bristol Composites Institute, School of Civil, Aerospace and Design Engineering, University of Bristol, Bristol, BS8 1TR, United Kingdom

[2]Research School of Chemistry, Australian National University, Canberra ACT 2601, Australia; Email: valeska.ting@anu.au

[3]School of Archaeological and Forensic Sciences, University of Bradford, Bradford, BD7 1DP, United Kingdom

[4]School of Chemistry, University of Bristol, Bristol, BS8 1TR, United Kingdom

[5]Polymer IRC, School of Engineering, University of Bradford, Bradford, BD7 1DP, United Kingdom

[6]Bristol Composites Institute, School of Electrical, Electronic and Mechanical Engineering, University of Bristol, Bristol, BS8 1TR, United Kingdom

* Email: s.nayak@bristol.ac.uk ; valeska.ting@anu.au

† These authors have contributed equally to this work.

No of pages: 32

No of Figures: 29

No of Tables: 1

Images of some of the extrudates and their PCL composite sheet after hot press

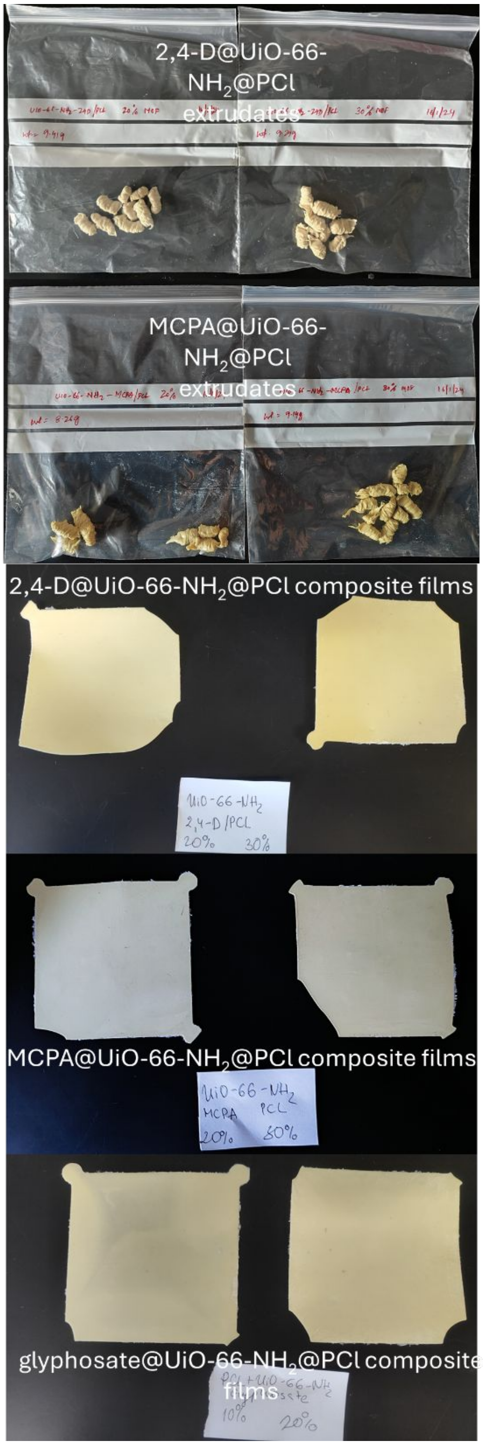

Figure S1. Pictures of some of the PCL composites prepared via twin screw extrusion methods (the size of the composite sheets are roughly 8 x 8 cm)

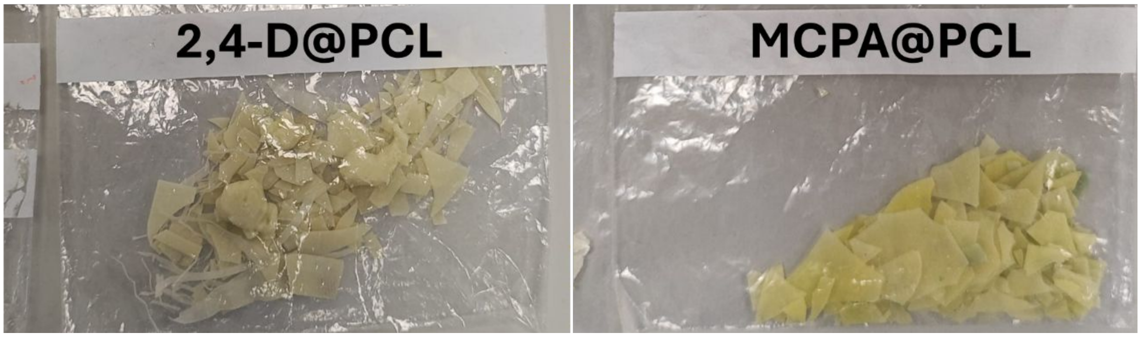

Figure S2. Brittle transformation of PCL composites observed within a few days when herbicides were directly loaded into the PCL matrix.

PXRD Analysis

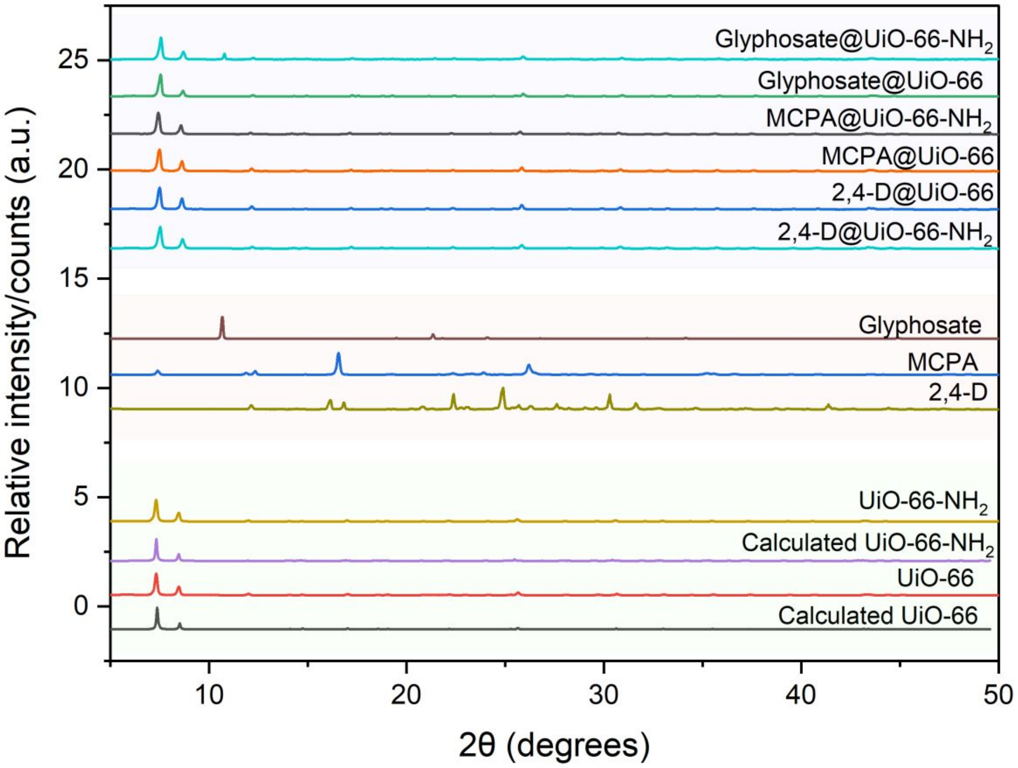

Figure S3. PXRD patterns of pristine UiO-66 and UiO-66-NH_2 , 2,4-D, MCPA, glyphosate and 2,4-D-, MCPA-, glyphosate- loaded UiO-66 and UiO-66-NH_2

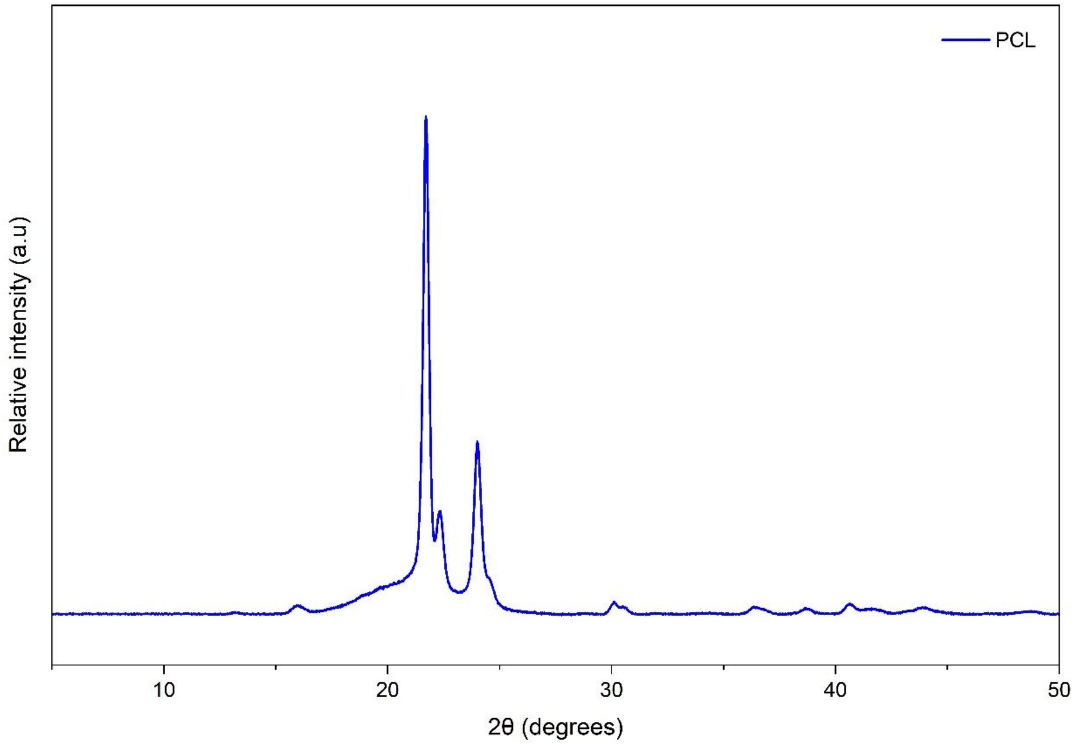

Figure S4. PXRD patterns of PCL

FTIR analysis

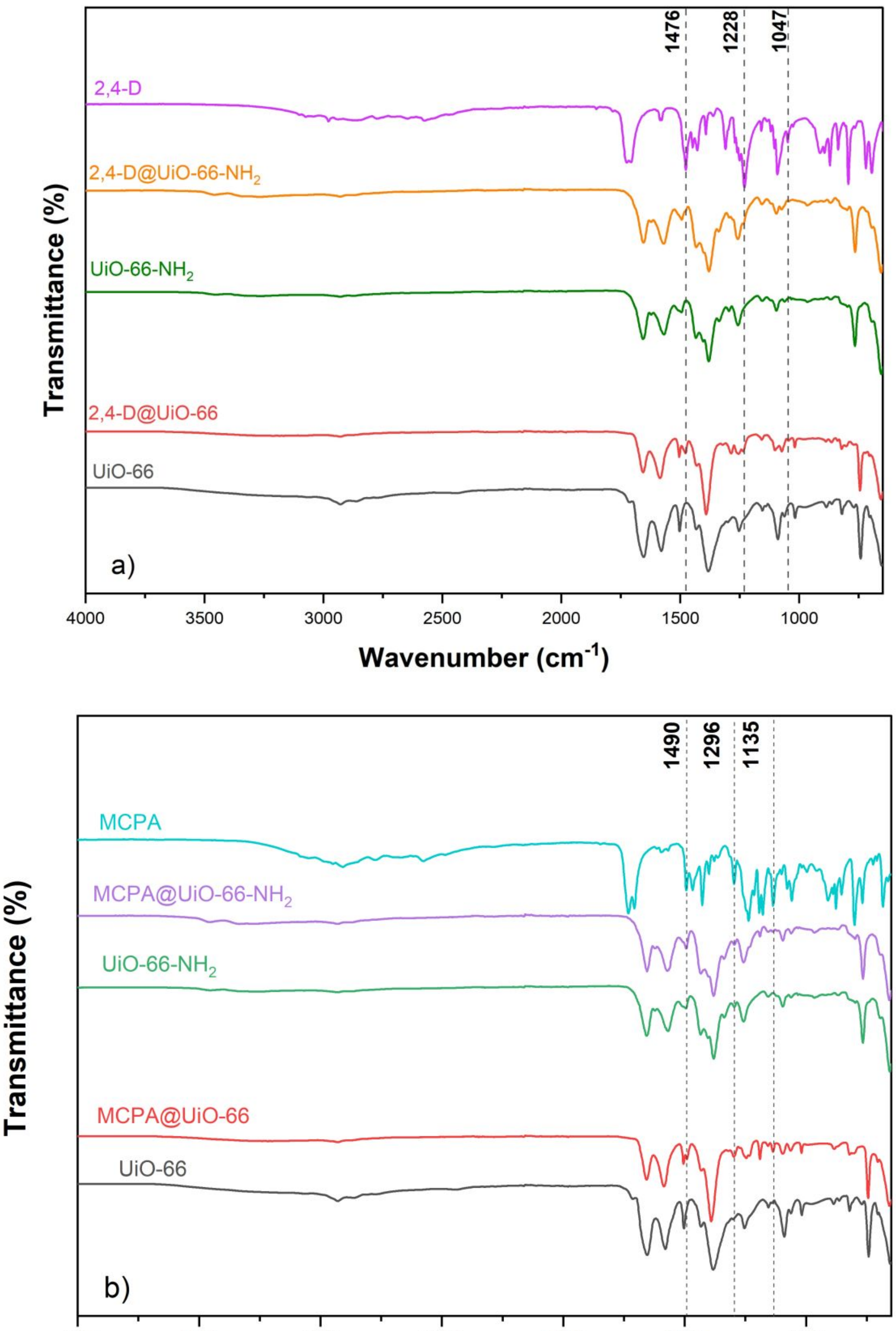

1476
1228
1047
2,4-D
2,4-D@UiO-66-NH2
UiO-66-NH2
2,4-D@UiO-66
UiO-66
a)
Transmittance (%)
4000
3500
3000
2500
2000
1500
1000
Wavenumber (cm-1)
1490
1296
1135
MCPA
MCPA@UiO-66-NH2
UiO-66-NH2
MCPA@UiO-66
UiO-66
b)
Transmittance (%)
4000
3500
3000
2500
2000
1500
1000
Wavenumber (cm-1)

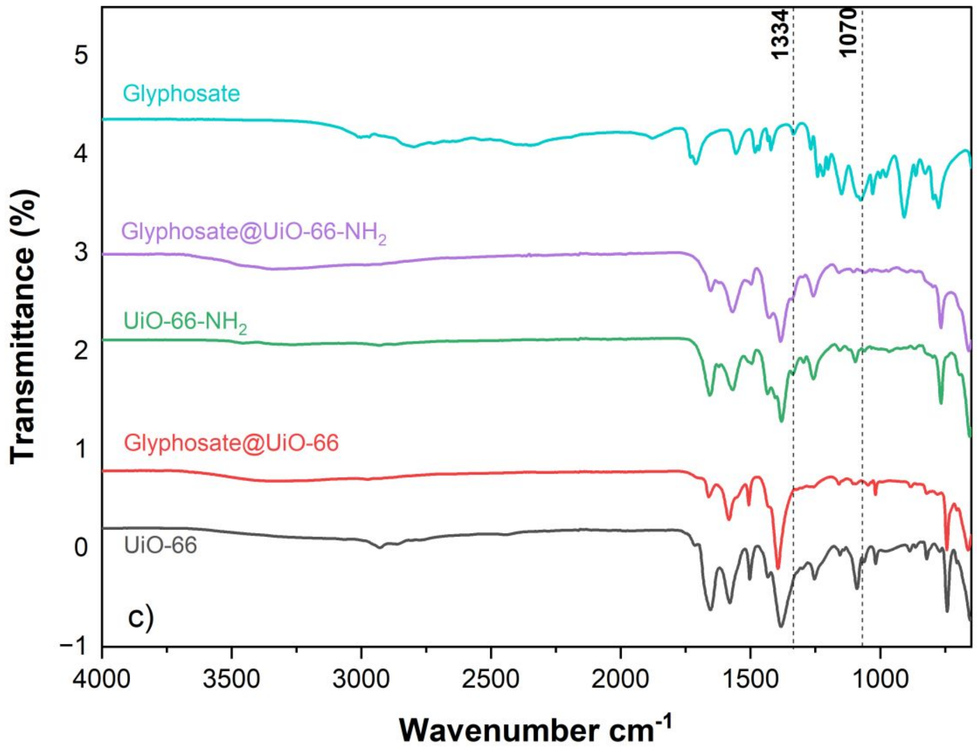

Figure S5. FTIR spectra of a) UiO-66, UiO-66-NH_2 loaded with 2,4-D, and 2,4-D b) UiO-66, UiO-66-NH_2 loaded with MCPA, and MCPA and c) UiO-66, UiO-66-NH_2 loaded with glyphosate, and glyphosate.

Thermogravimetric Analysis

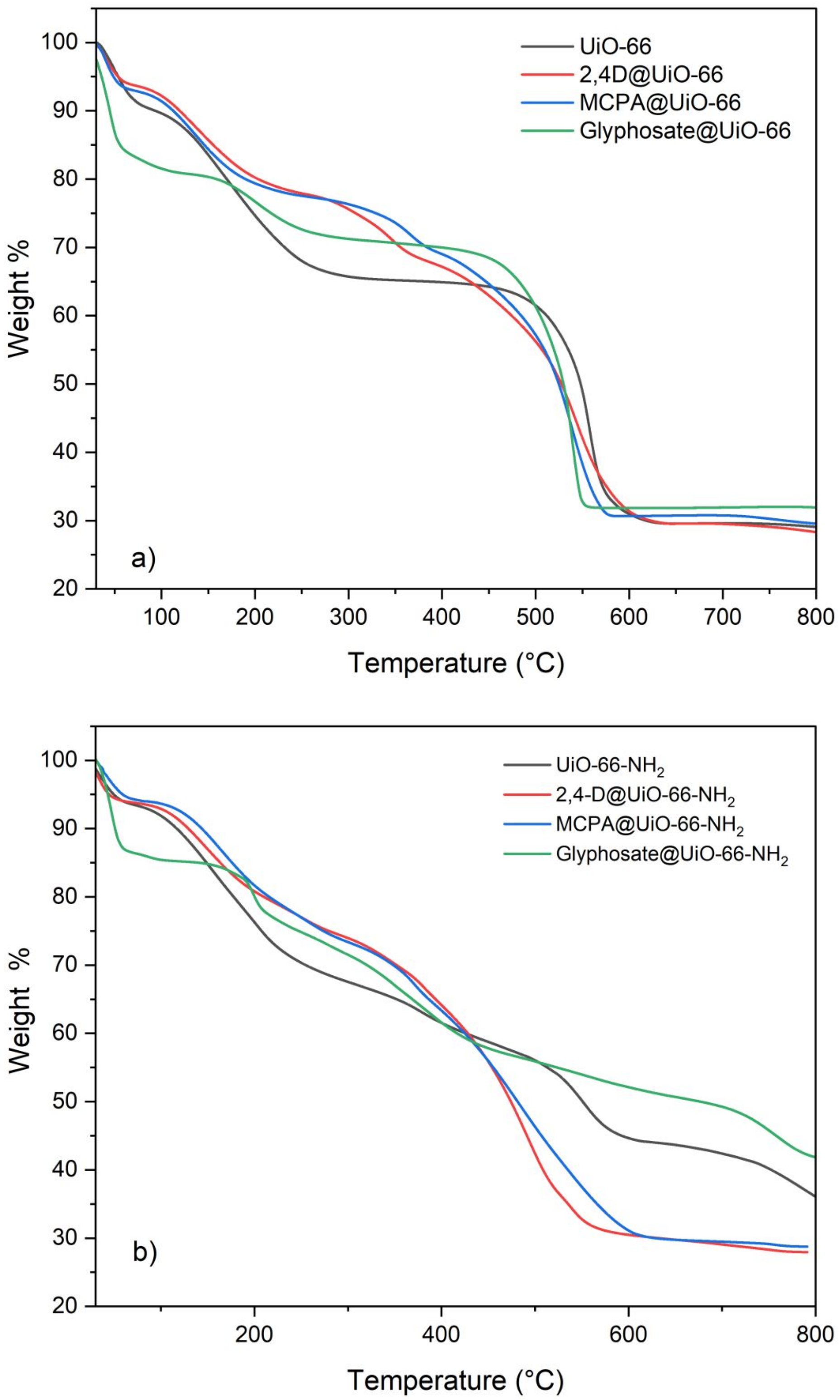

Figure S6. a) UiO-66 and herbicide@UiO-66; b) UiO-66-NH_2 and herbicide@UiO-66-NH_2

SEM and Elemental mapping studies

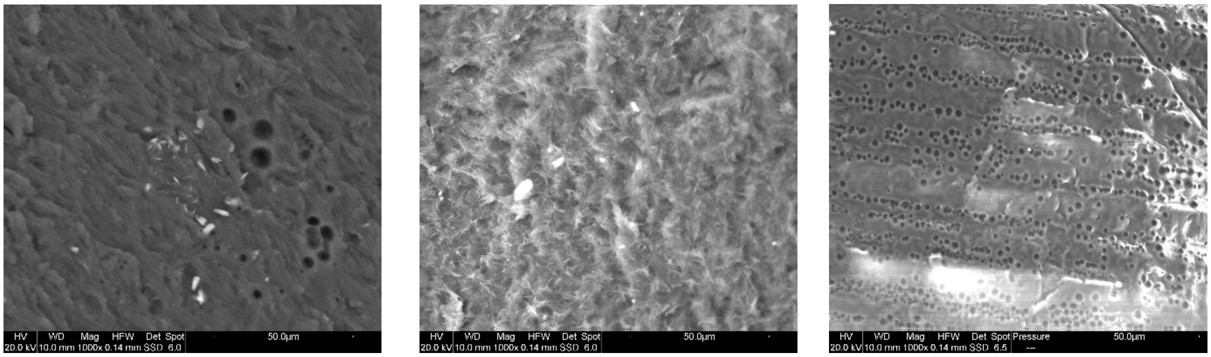

(a) (b) (c)

Figure S7. SEM images of PCL composites with direct incorporation of (a) 2,4-D, (b) MCPA and (d) glyphosate.

2,4-D@PCL

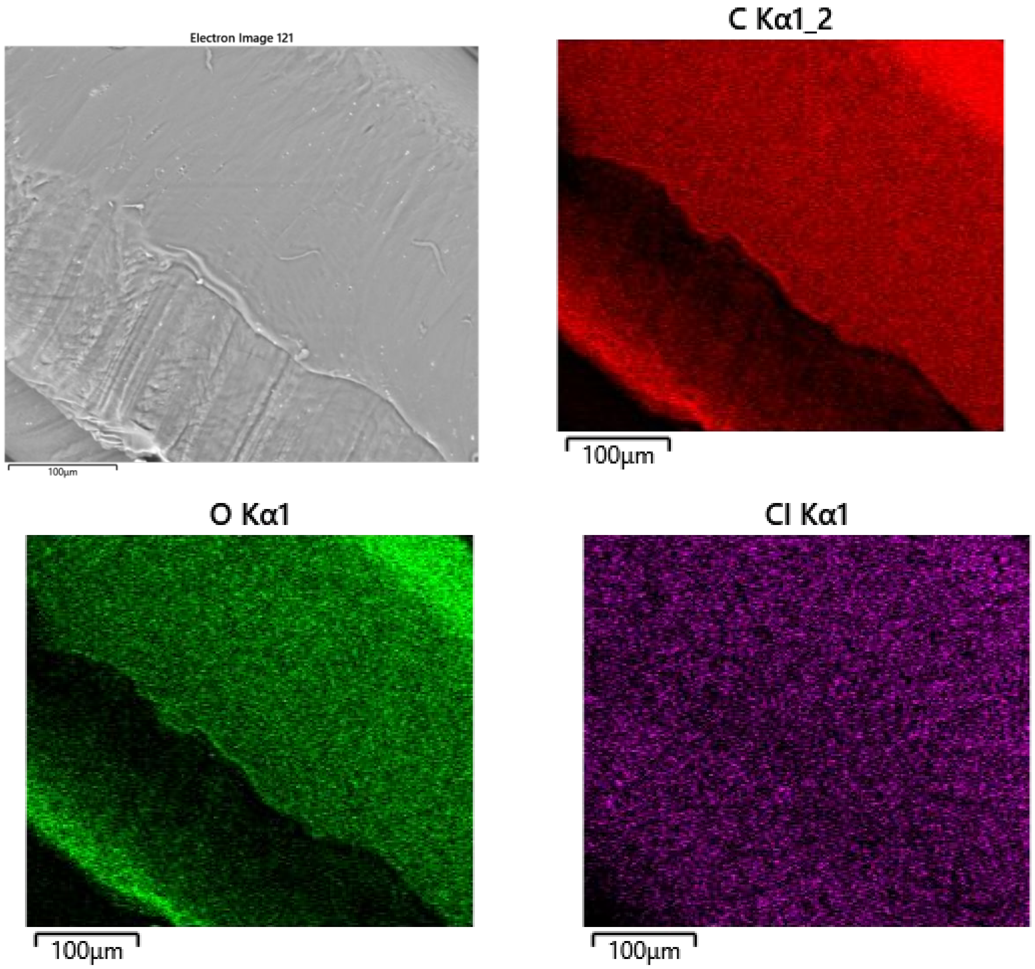

Figure S8. Elemental map of 2,4-D@PCL

MCPA@PCL

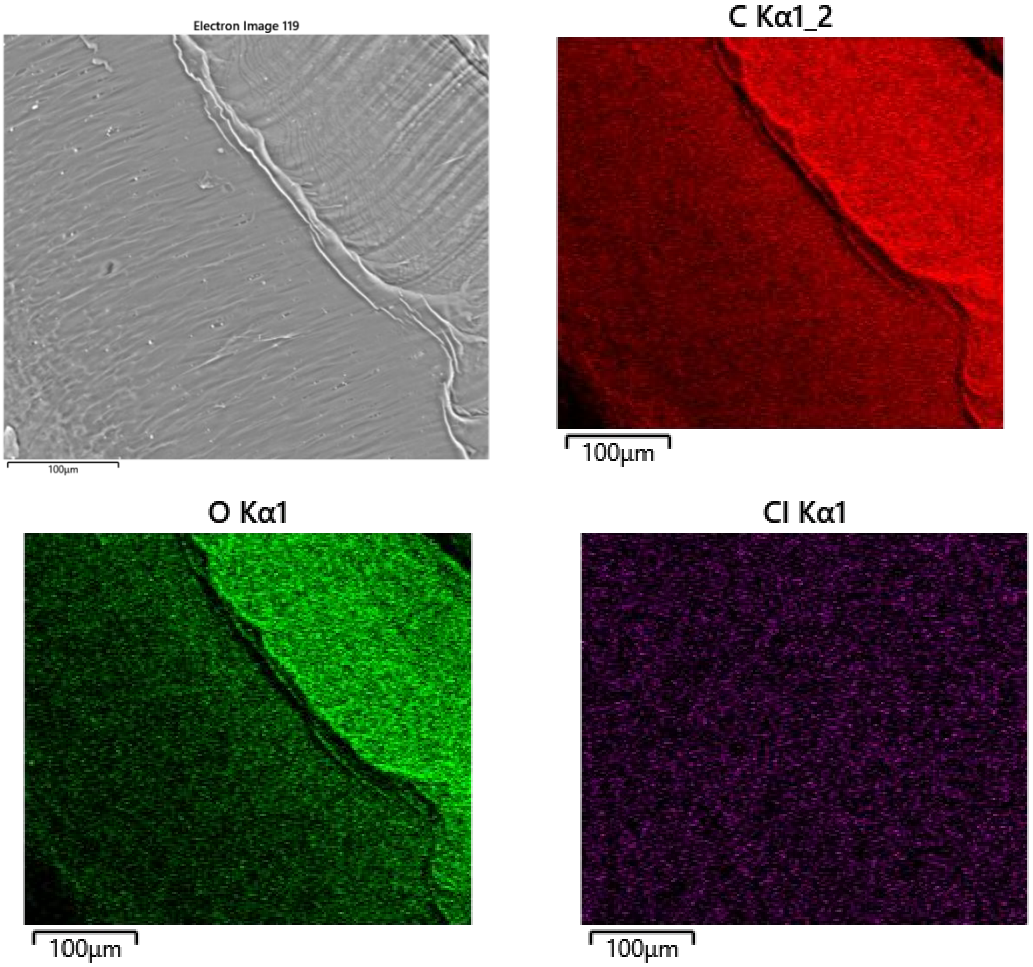

Figure S9. Elemental map of MCPA@PCL

Glyphosate@PCL

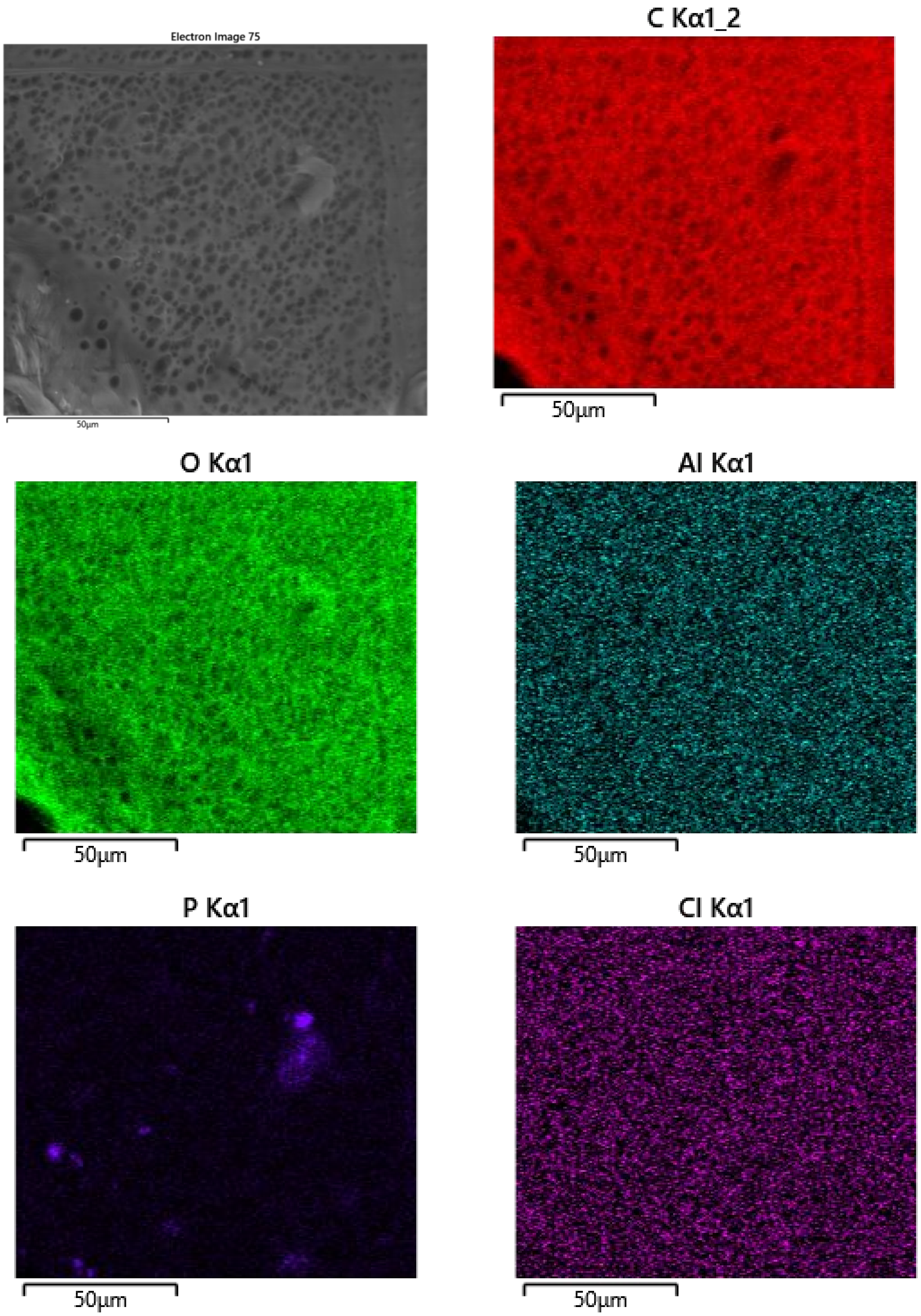

Figure S10. Elemental map of glyphosate@PCL

2,4-D@UiO-66@PCL (10%)

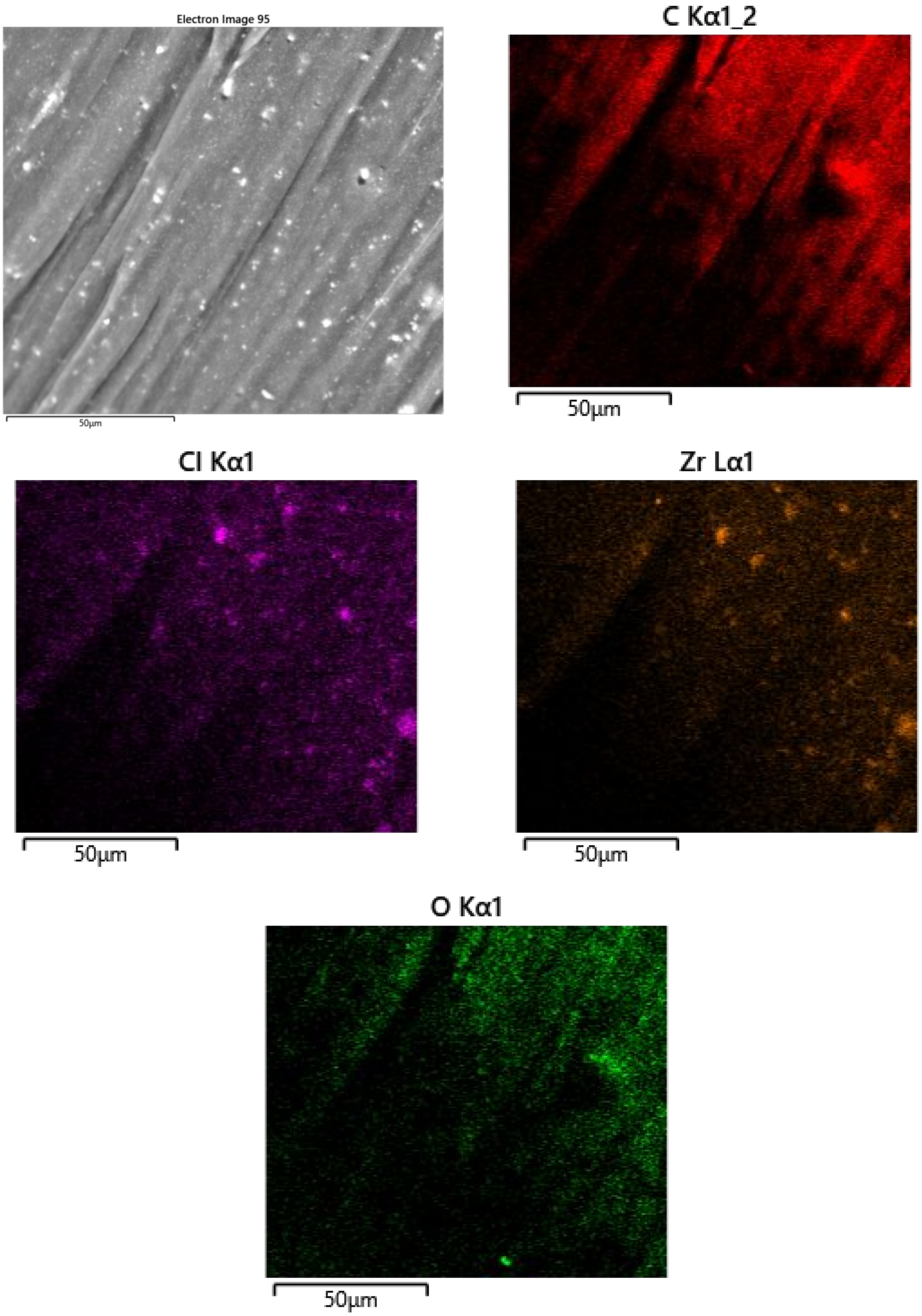

Figure S11. Elemental map of 2,4-D@UiO-66@PCL (10%)

2,4-D@UiO-66-NH_2@PCL (10%)

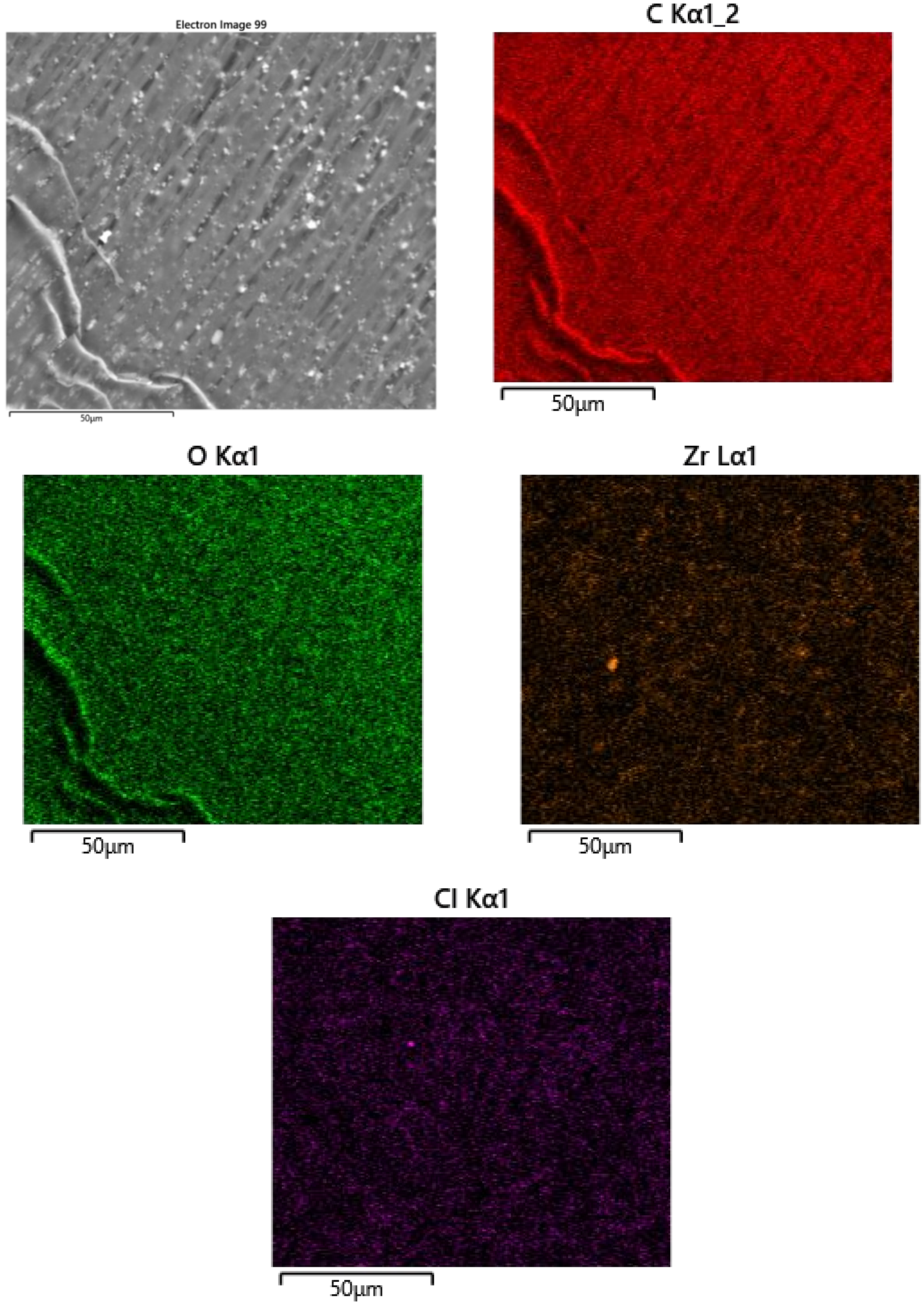

Figure S12. Elemental map of 2,4-D@UiO-66-NH_2@PCL (10%)

MCPA@UiO-66@PCL (10%)

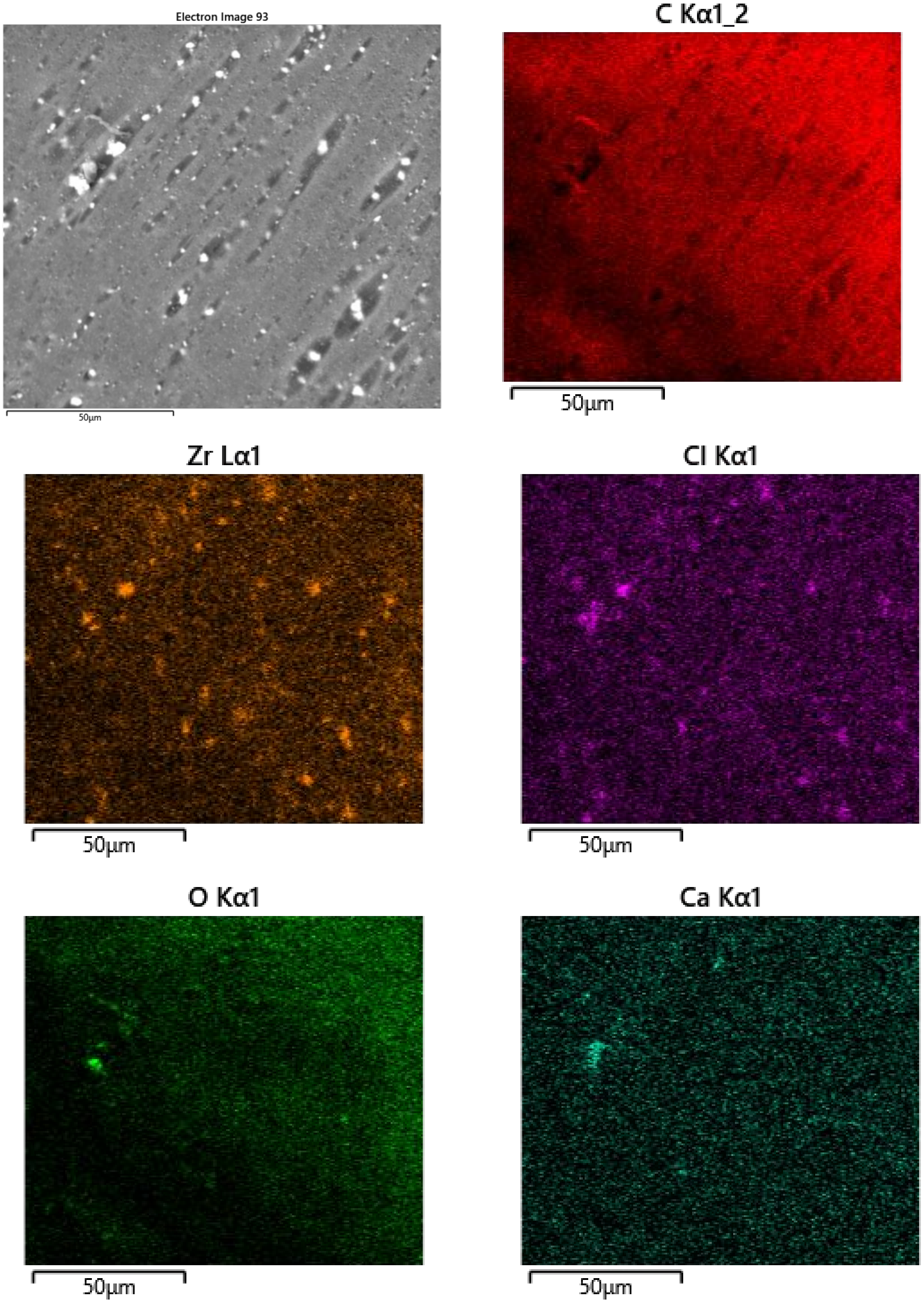

Figure S13. Elemental map of MCPA@UiO-66-NH_2@PCL (10%)

MCPA@UiO-66-NH_2@PCL (10%)

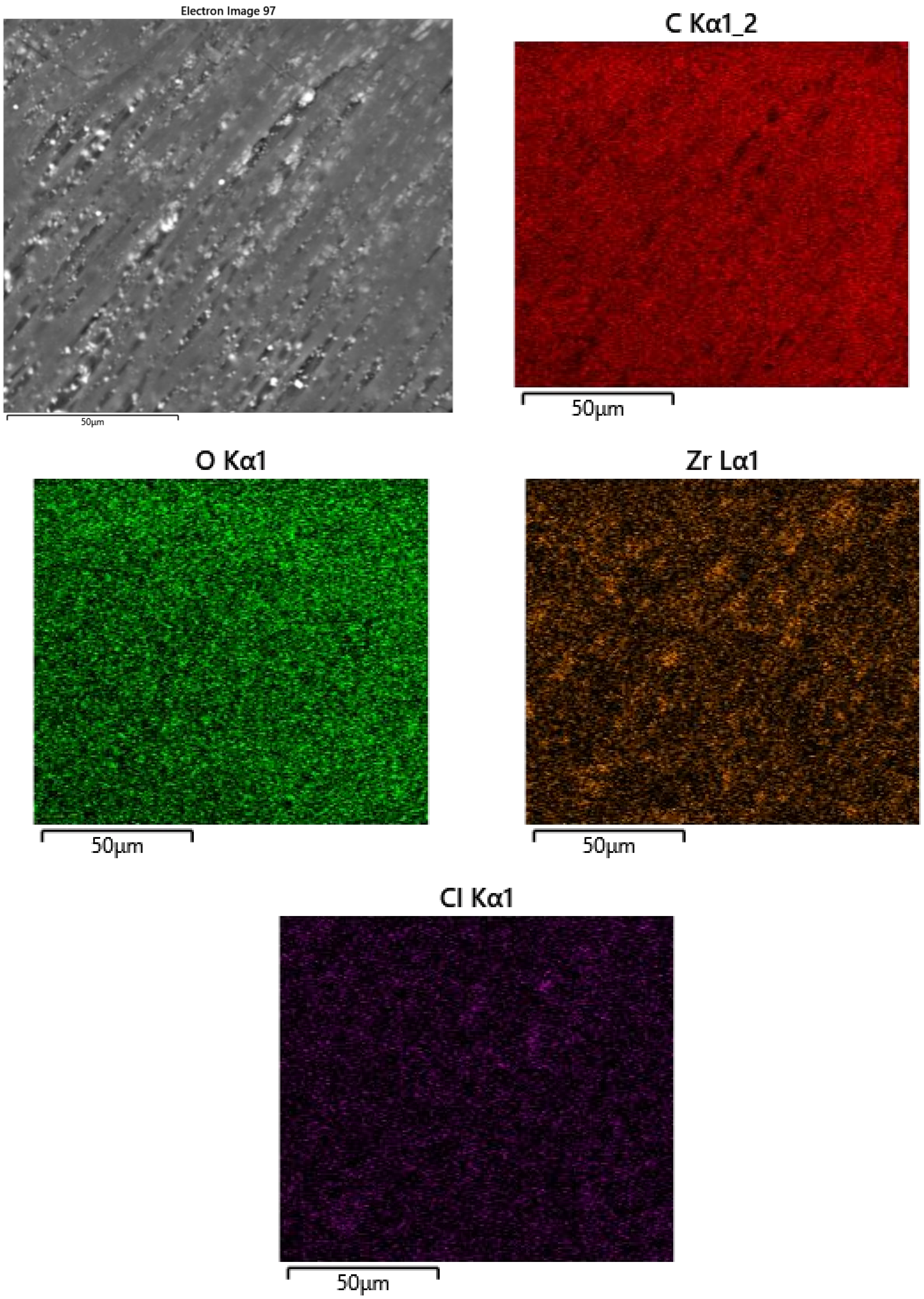

Figure S14. Elemental map of MCPA@UiO-66-NH_2@PCL (10%)

Glyphosate@UiO-66@PCL (10%)

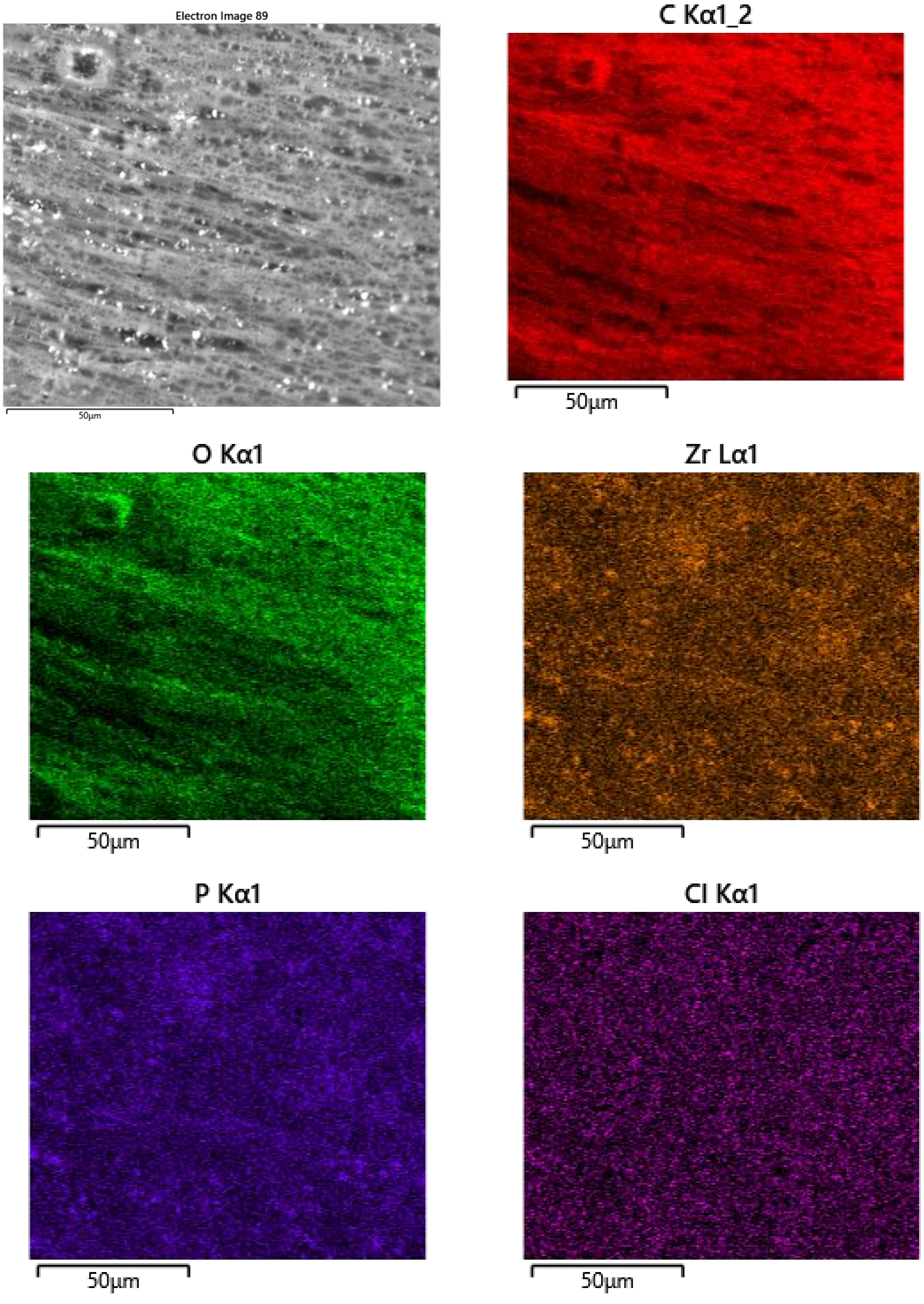

Figure S15. Elemental map of glyphosate@UiO-66@PCL (10%)

Glyphosate@UiO-66-NH_2@PCL (10%)

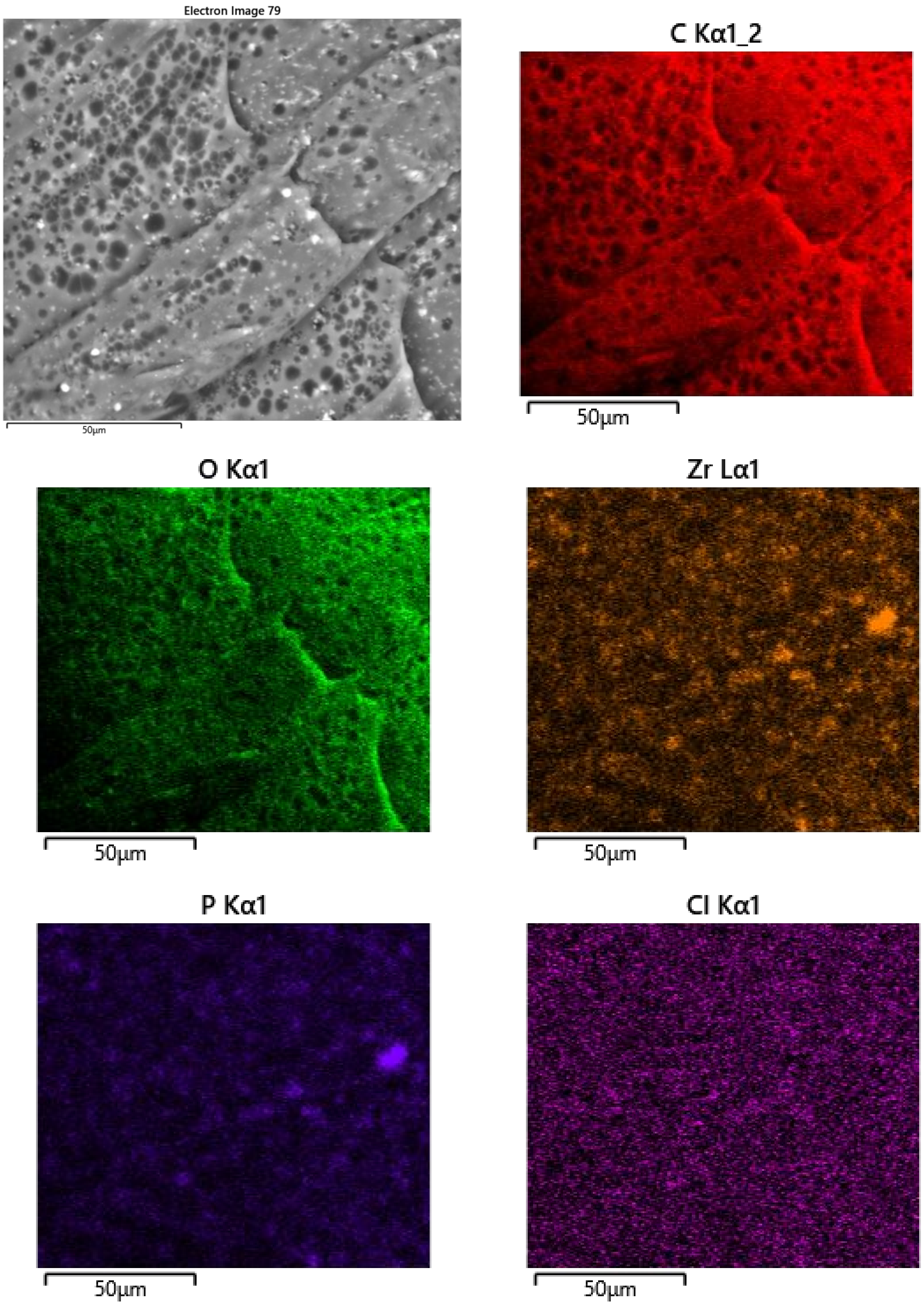

Figure S16. Elemental map of Glyphosate@UiO-66-NH_2@PCL (10%)

2,4-D@UiO-66@PCL (20%)

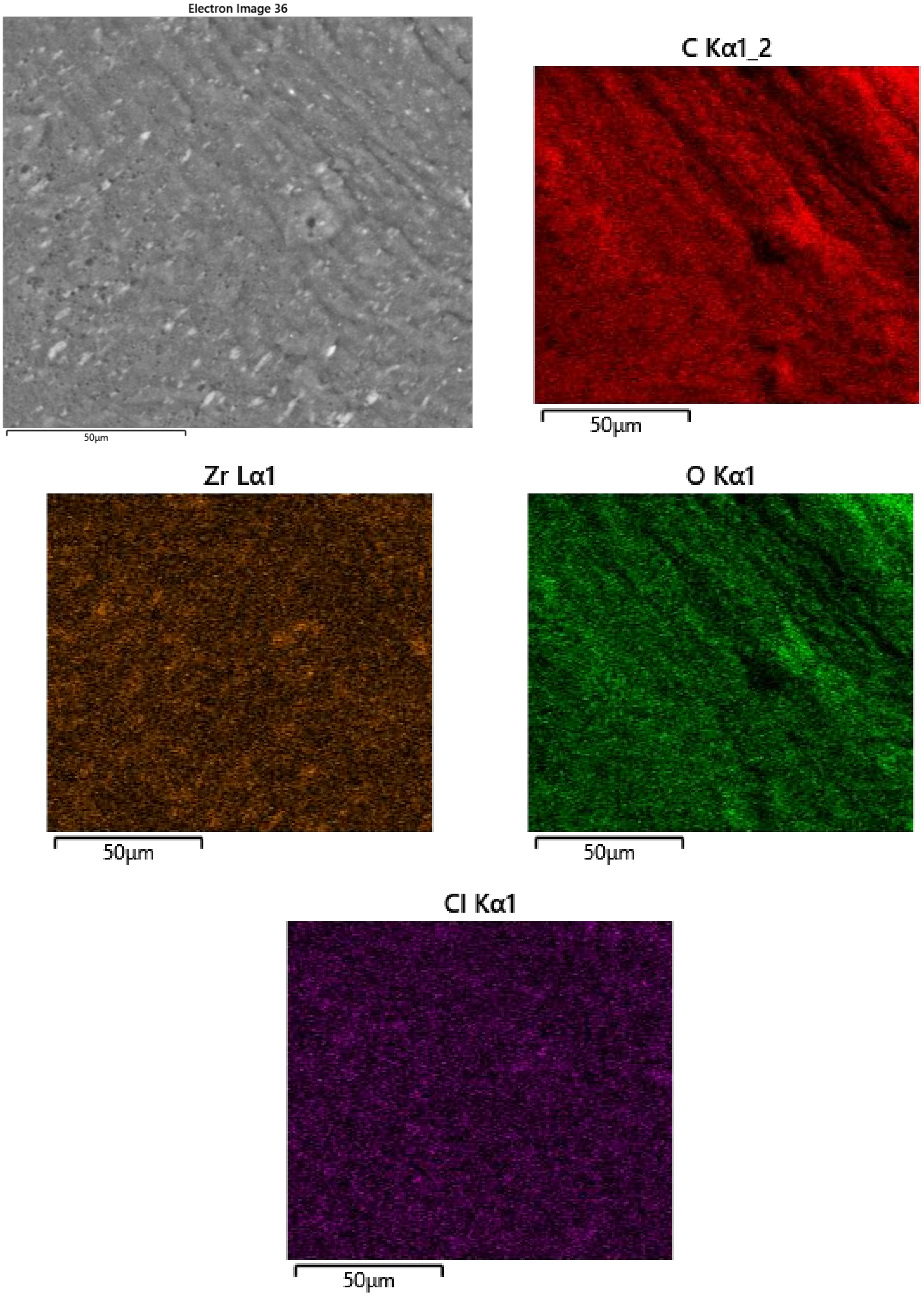

Figure S17. Elemental map of 2,4-D@UiO-66@PCL (20%)

2,4-D@UiO-66-NH_2@PCL (20%)

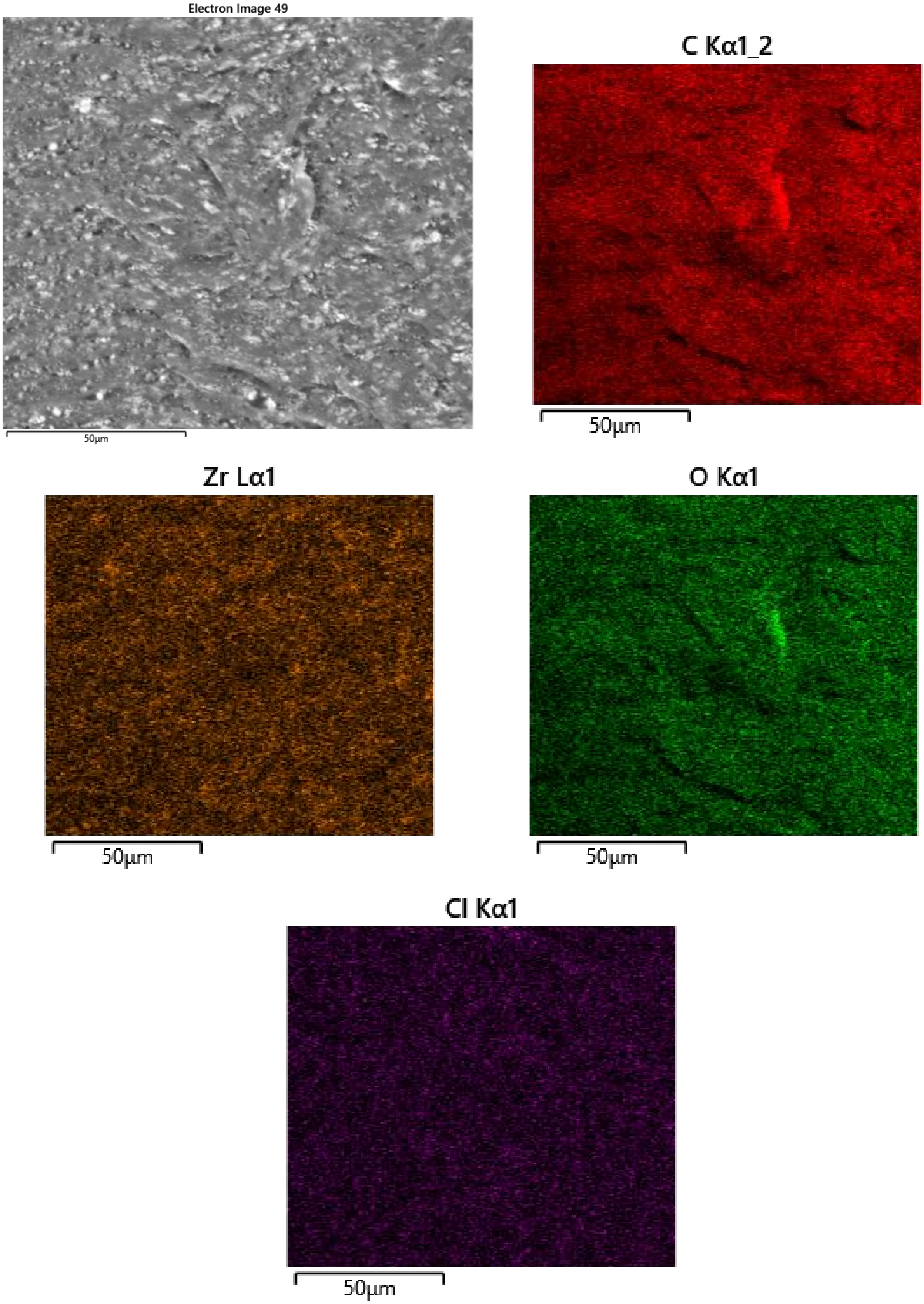

Figure S18. Elemental map of 2,4-D@UiO-66-NH_2@PCL (20%)

MCPA@UiO-66@PCL (20%)

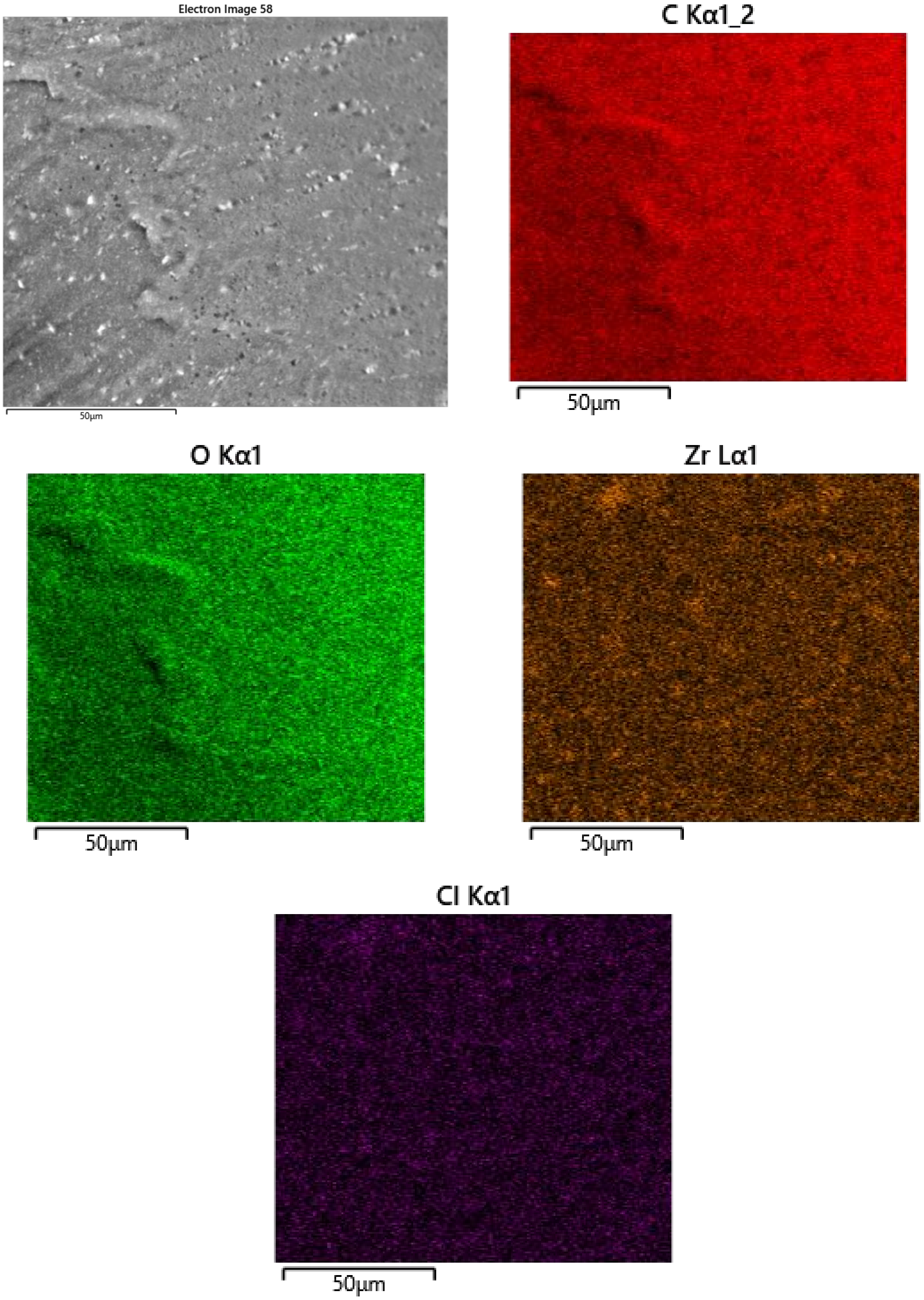

Figure S19. Elemental map of MCPA@UiO-66@PCL (20%)

MCPA@UiO-66-NH$_2$@PCL (20%)

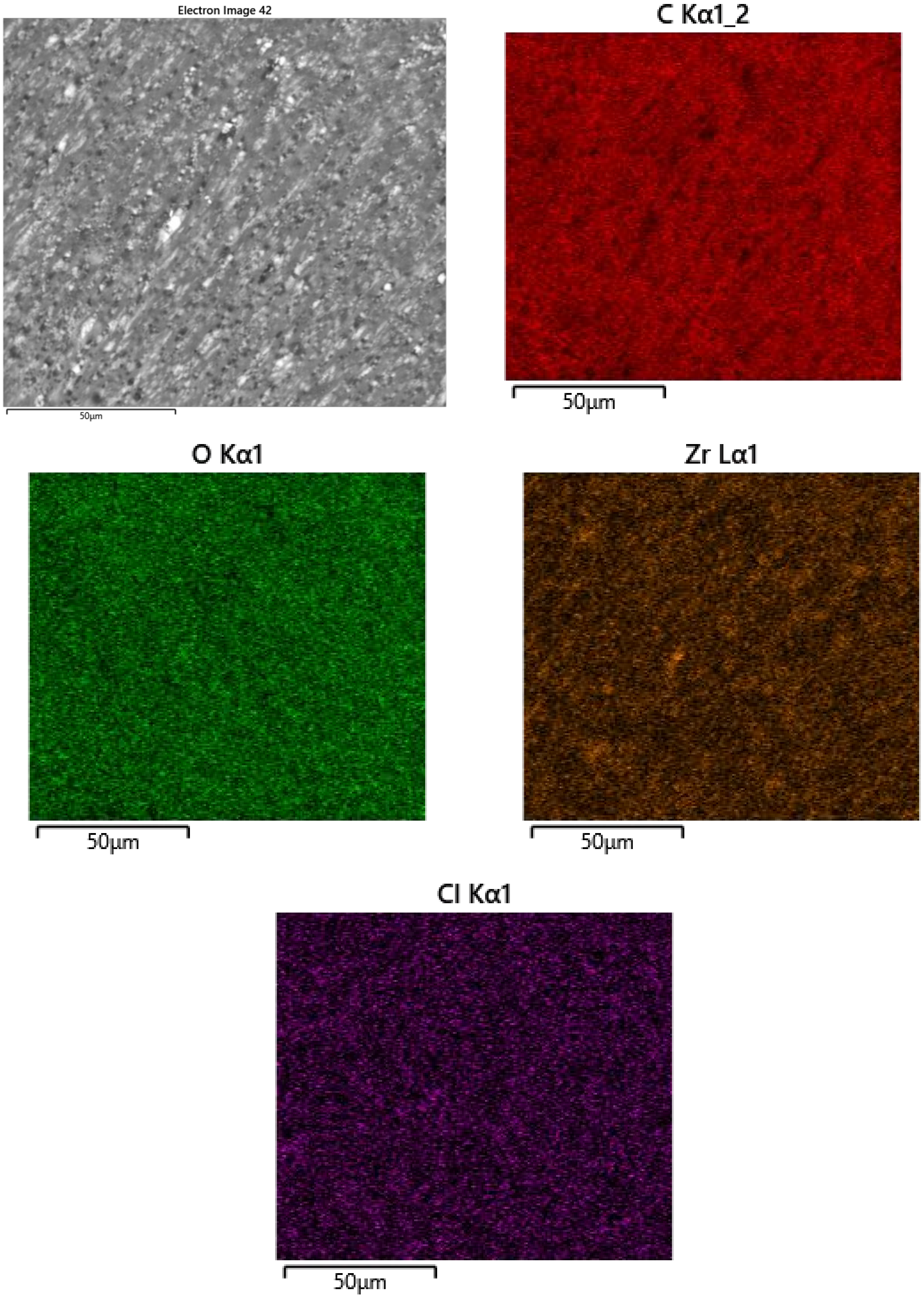

Figure S20. Elemental map of MCPA@UiO-66-NH$_2$@PCL (20%)

Glyphosate@UiO-66@PCL (20%)

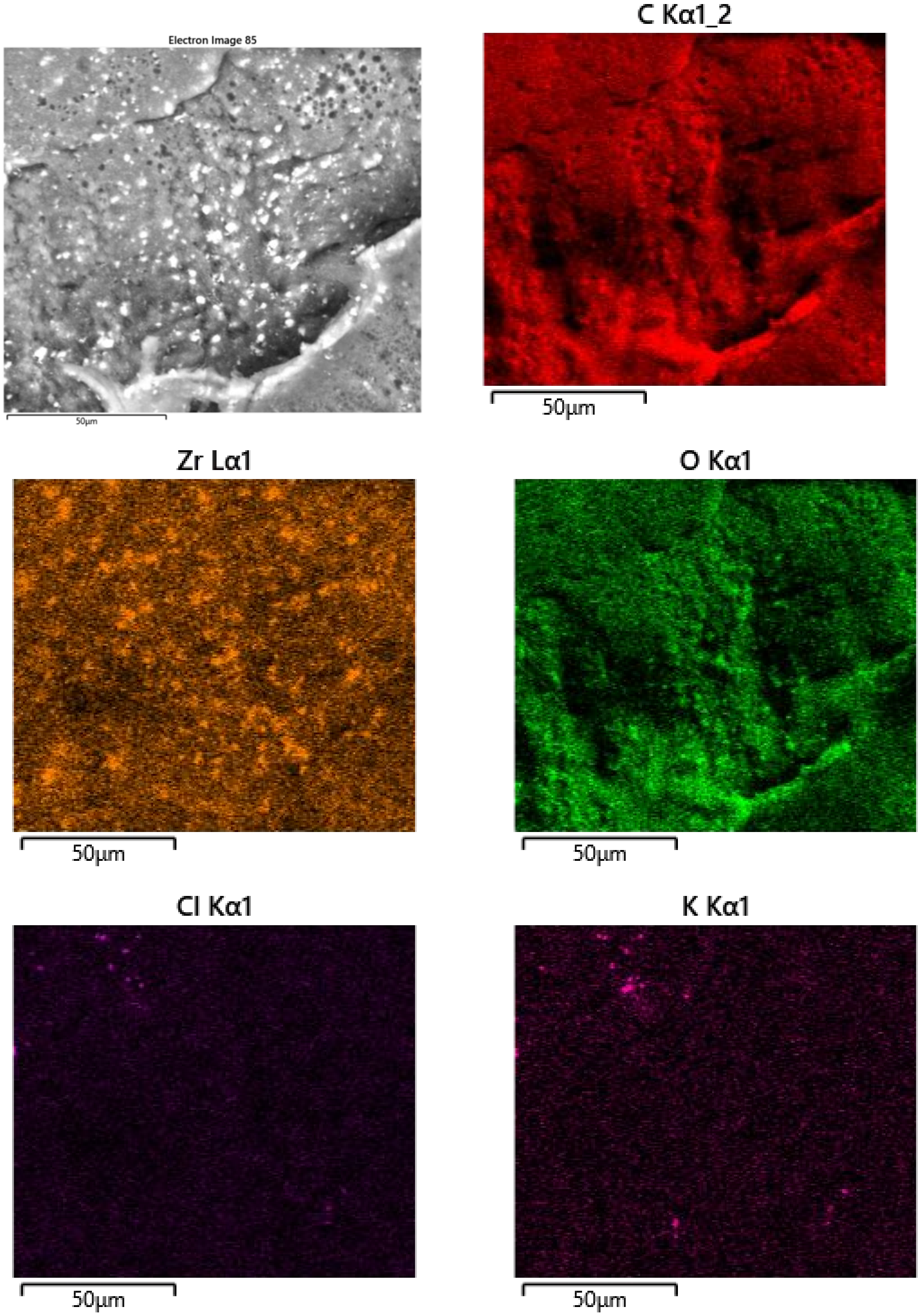

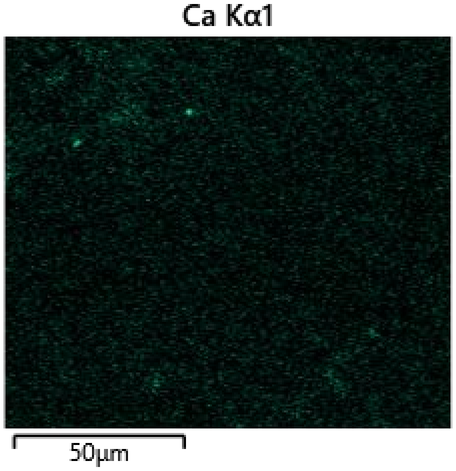

Figure S21. Elemental map of glyphosate@UiO-66@PCL (20%)

Glyphosate@UiO-66-NH_2@PCL (20%)

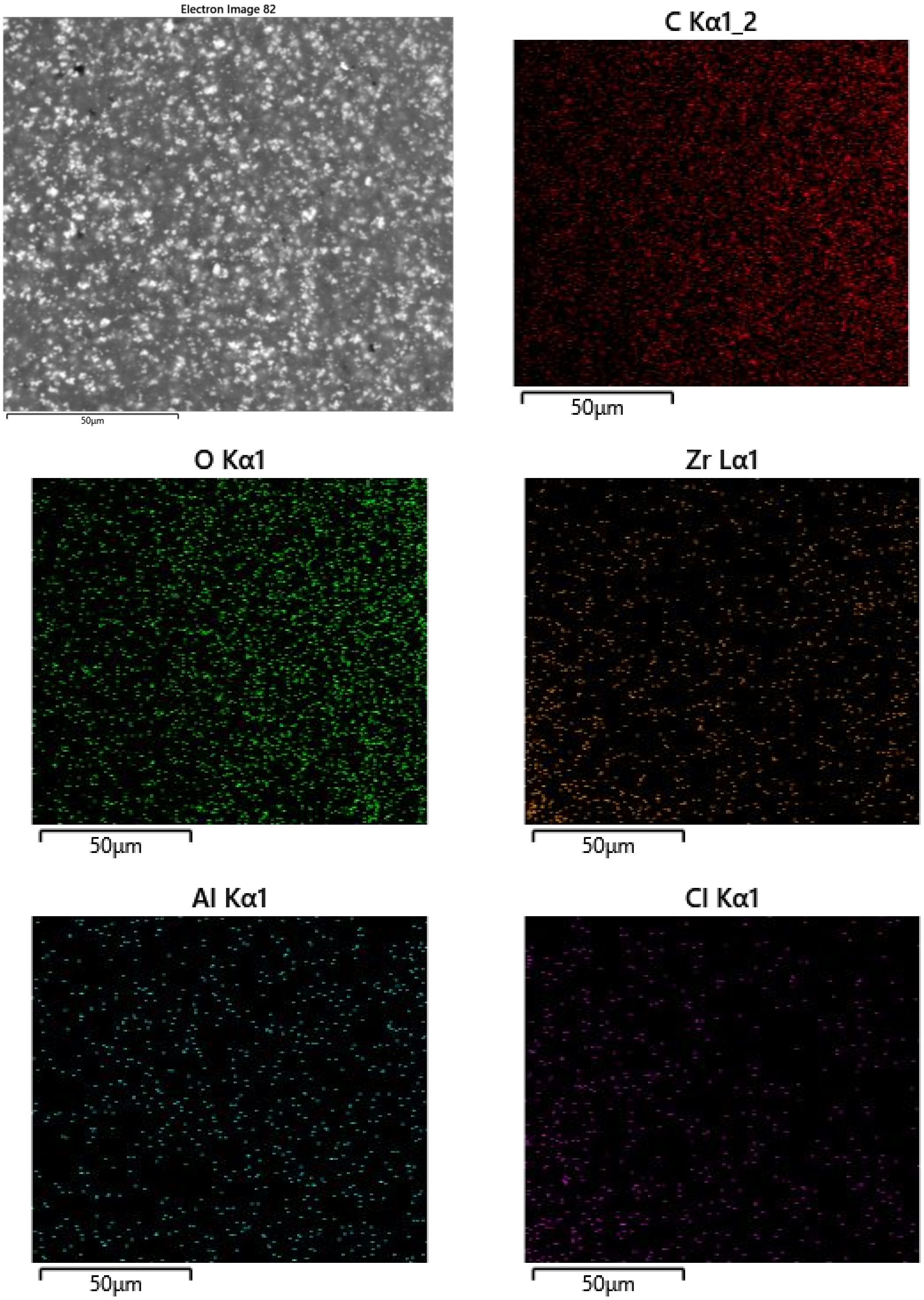

Figure S22. Elemental map of glyphosate@UiO-66-NH_2@PCL (20%)

2,4-D @UiO-66@PCL (30%)

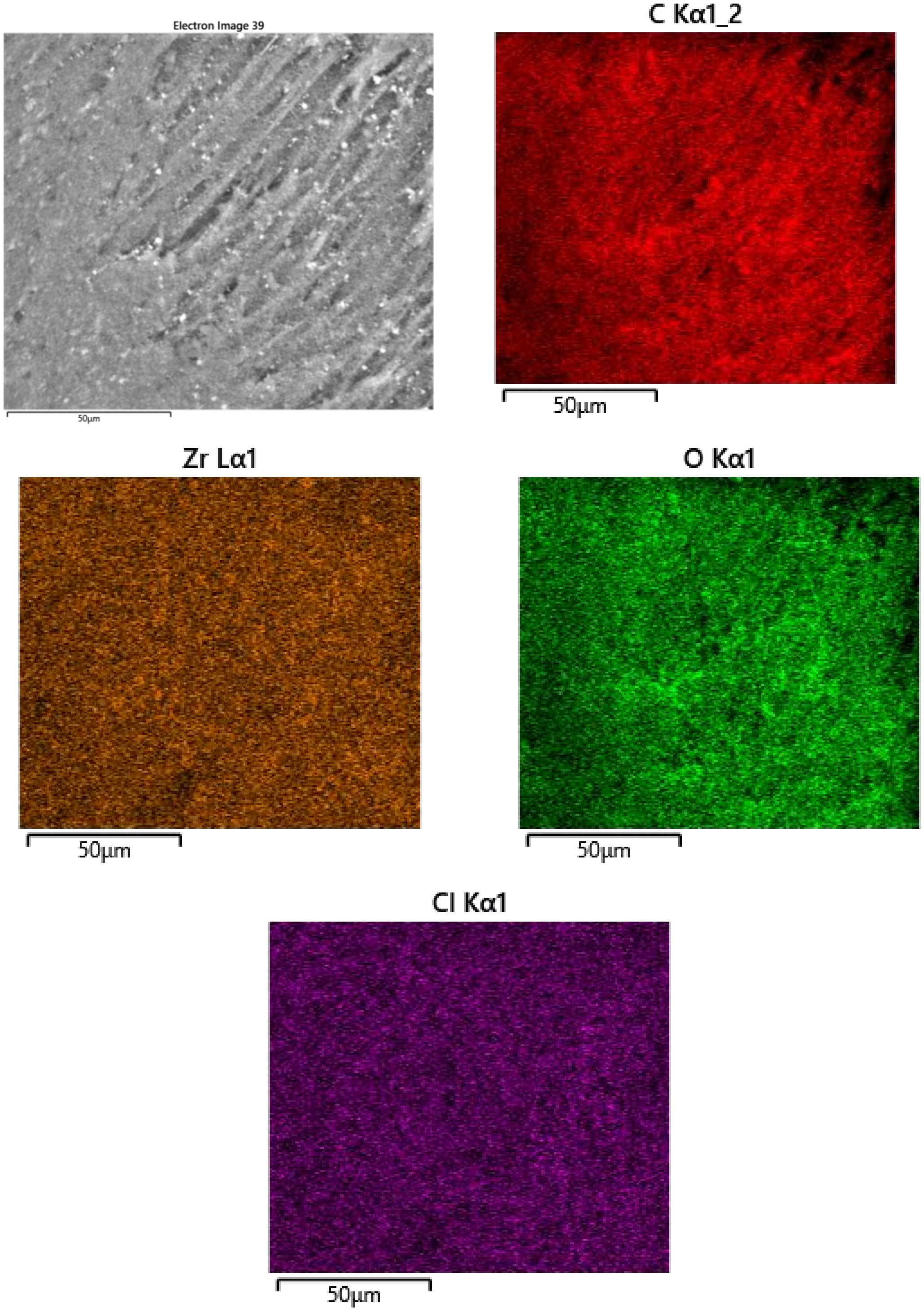

Figure S23. Elemental map of 2,4-D @UiO-66@PCL (30%)

2,4-D@UiO-66-NH_2@PCL (30%)

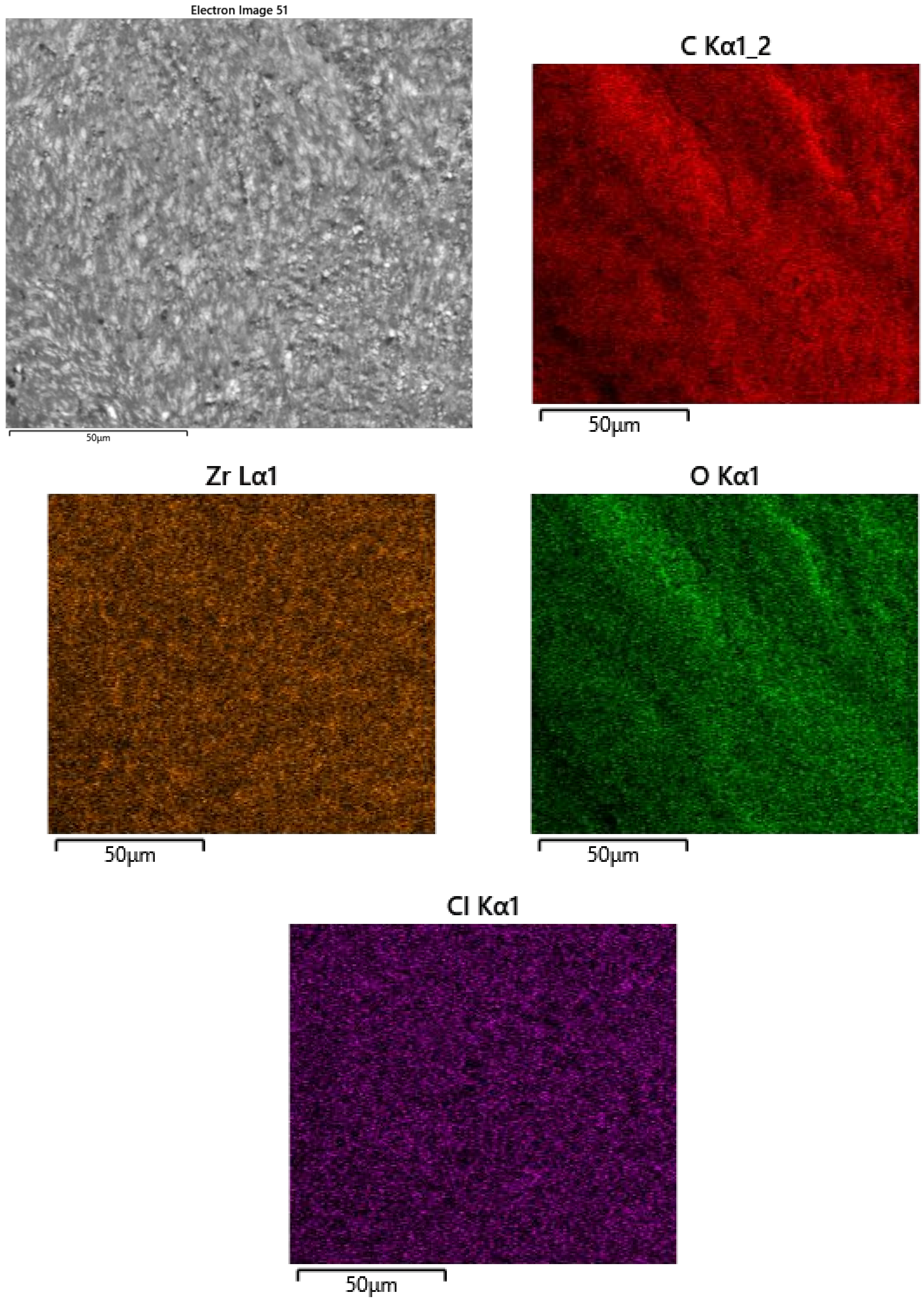

Figure S24. Elemental map of 2,4-D@UiO-66-NH_2@PCL (30%)

MCPA@UiO-66@PCL (30%)

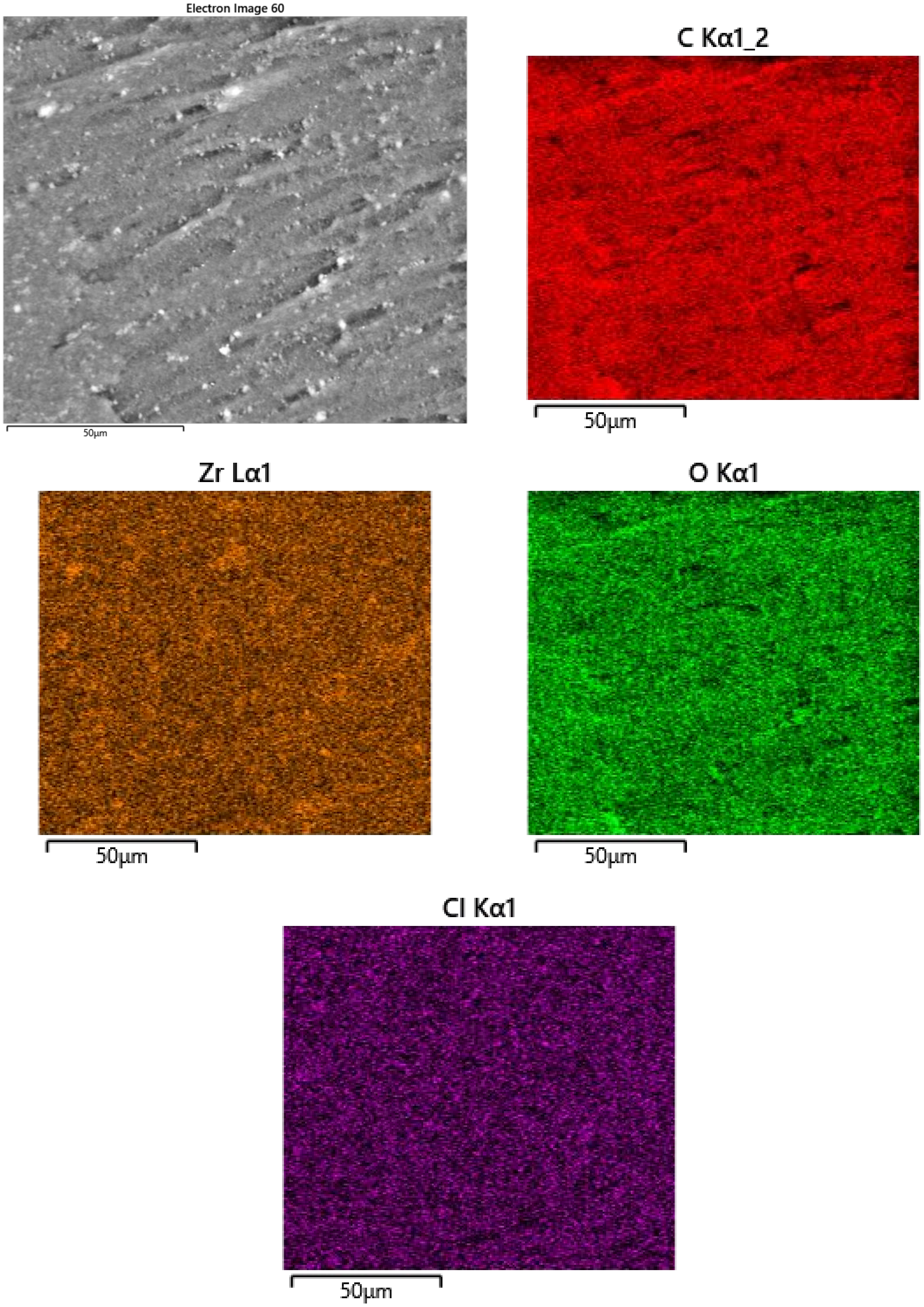

Figure S25. Elemental map of MCPA@UiO-66@PCL (30%)

MCPA@UiO-66-NH_2@PCL (30%)

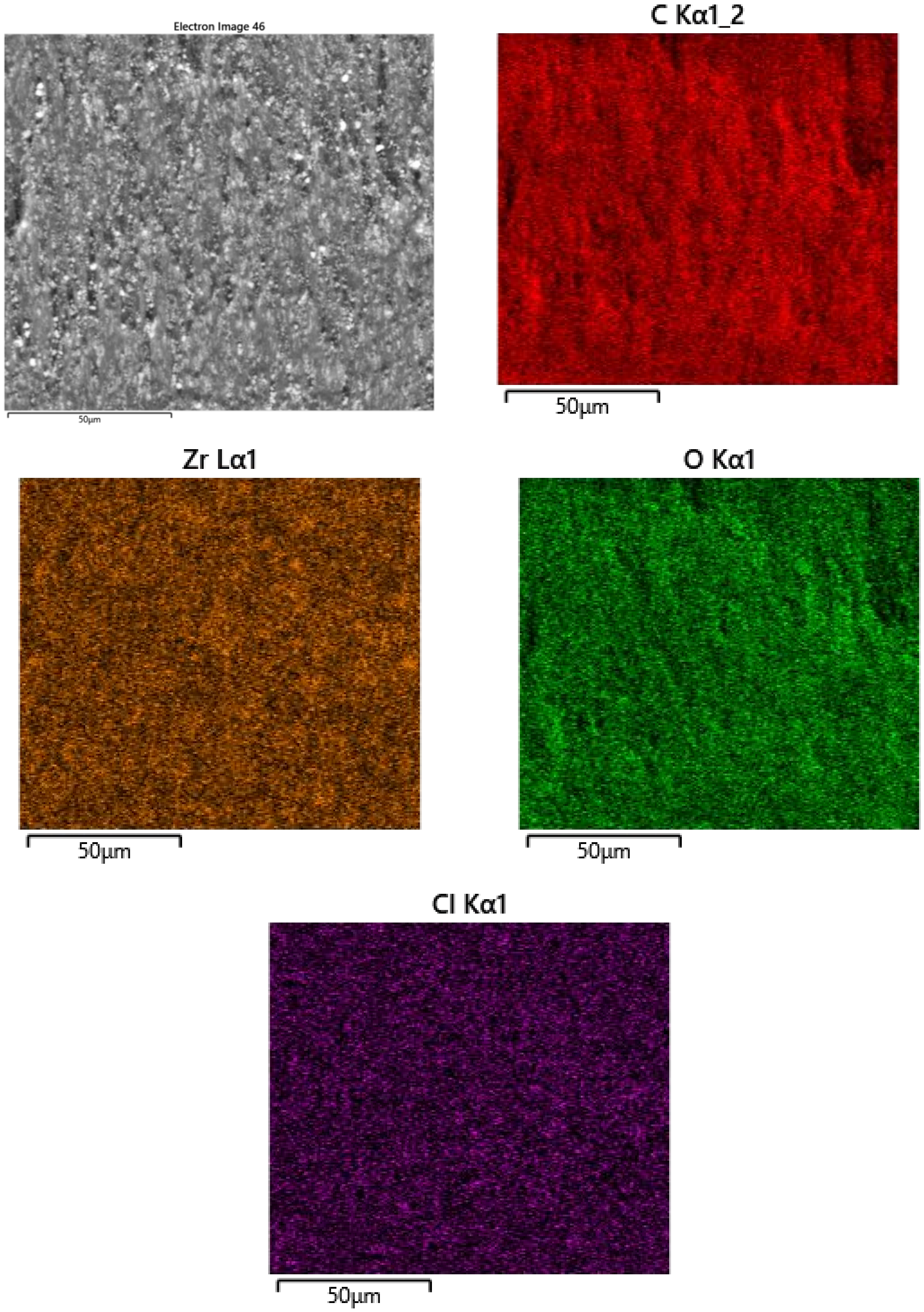

Figure S26. Elemental map of MCPA@UiO-66-NH_2@PCL (30%)

Glyphosate@UiO-66@PCL (30%)

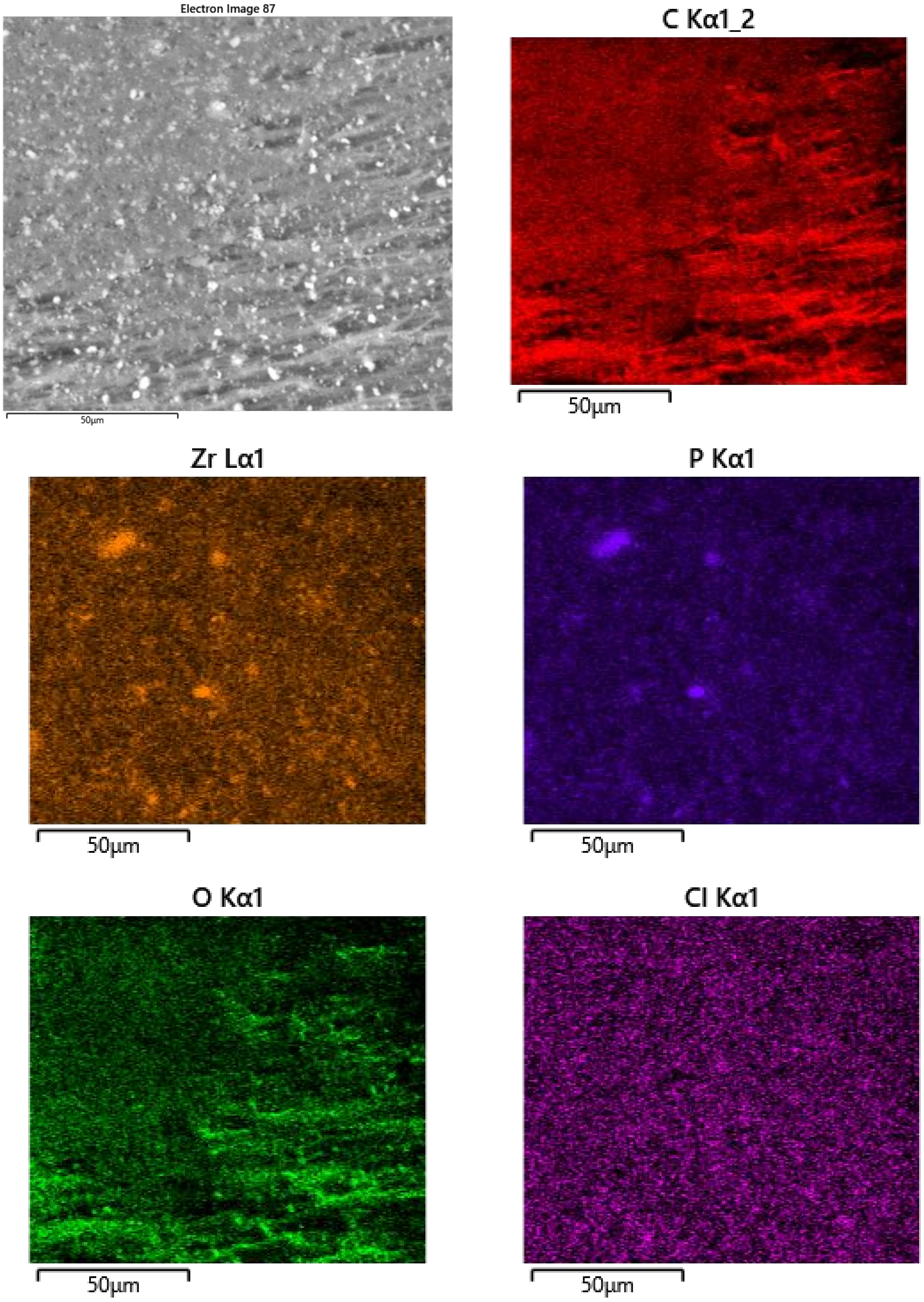

Figure S27. Elemental map of glyphosate@UiO-66@PCL (30%)

Glyphosate@UiO-66-NH_2@PCL (30%)

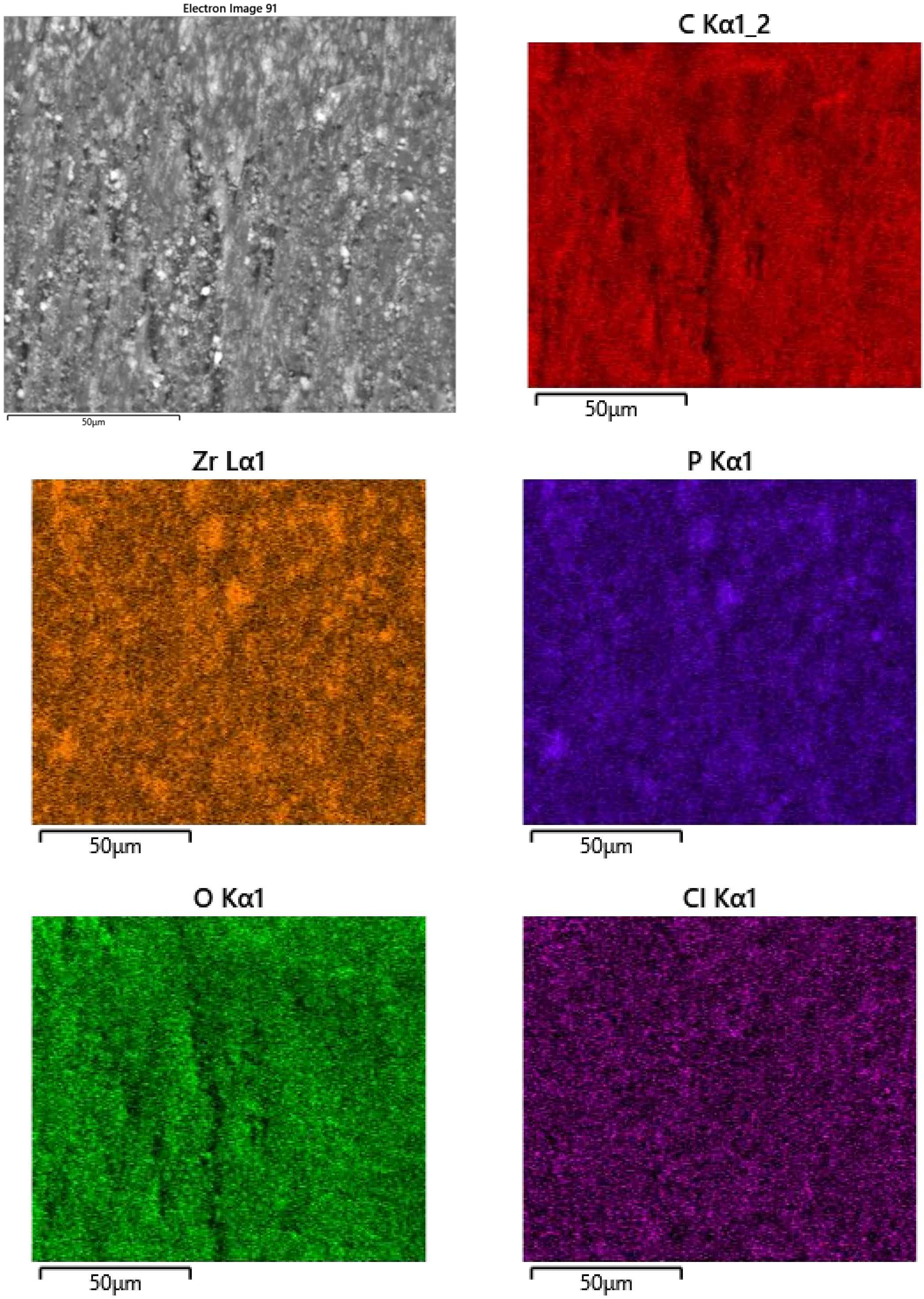

Figure S28. Elemental map of glyphosate@UiO-66-NH_2@PCL (30%)

Table S1. Young's Modulus of PCL composites												
Herbicide @MOFs	Young's Modulus	error	Young's Modulus	error	Young's Modulus	error	Young's Modulus	error	Young's Modulus	error	Young's Modulus	error
%	MPa	(±)	MPa	(±)	MPa	(±)	MPa	(±)	MPa	(±)	MPa	(±)
	PCL		2,4-D@PCL		MCPA@PCL		Glyphosate@PCL		UiO-66@PCL		UiO-66-NH_2@PCL	
10	606.890	8.220	252.283	18.384	255.267	17.245	452.870	50.920	431.980	30.775	499.920	23.618
20	--	--	--	--	--	--	--	--	583.274	69.272	597.290	194.564
30	--	--	--	--	--	--	--	--	873.986	56.217	745.616	26.473
	MCPA@ UiO66@PCL		MCPA@UiO-66-NH_2@PCL		2,4-D@ UiO-66@PCL		2,4-D@UiO-66-NH_2@PCL		Glyphosate@UiO-66@PCL		Glyphosate@UiO-66-NH_2@PCL	
10	704.280	33.256	683.997	9.637	686.043	21.110	719.873	17.439	492.883	8.602	553.230	18.343
20	783.357	61.765	928.337	27.436	680.833	2.627	836.650	8.820	710.637	83.270	632.943	27.434
30	1028.440	10.131	957.240	22.935	1046.733	34.947	1065.453	86.279	678.770	76.095	632.943	27.434

Loading capacity of MOFs

For determination of loading capacity of MOFs, 0.05 g of the herbicide-loaded MOFs was suspended in 3 mL of distilled water and sonicated for 60 min. After sonication, a 1 mL aliquot of the solution was taken out and diluted with 2 mL of water. The concentration was measured using a UV−vis spectrophotometer at 230 nm for 2,4-D, 227 nm for MCPA and 250 nm for glyphosate. The mass of herbicide released was determined from the volume, and the loading capacity in percentage was calculated using the following equation:

$$Loading\ capacity\ \% = \frac{amount\ of\ herbicide\ (g)}{mass\ of\ MOF\ (g)} X\ 100 \qquad Equation\ S1$$

Release Studies

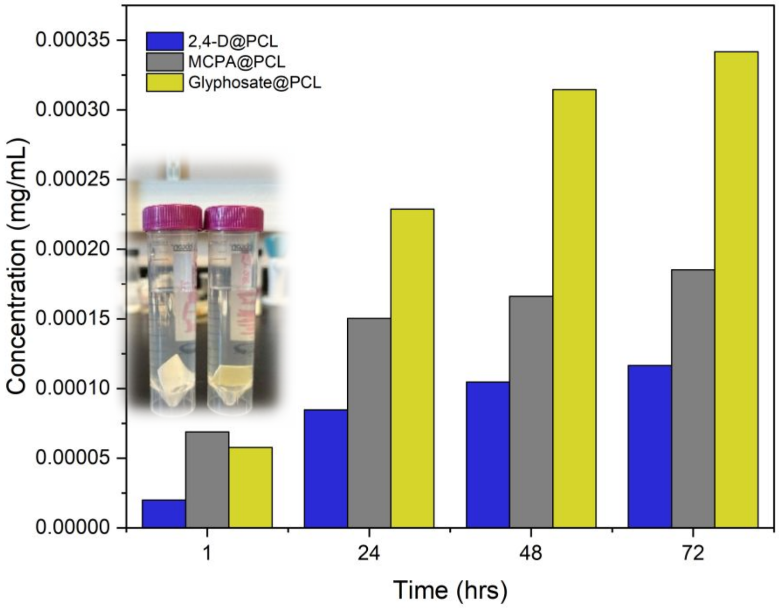

Figure S29. Release profile of herbicides@PCL (inlet showing the picture of the setup with 2 x2 cm piece of herbicide@PCL composite submerged in 30 mL of distilled water in a closed tube)
